# Chronic oxytocin-driven alternative splicing of Crfr2α induces anxiety

**DOI:** 10.1038/s41380-021-01141-x

**Published:** 2021-05-25

**Authors:** Julia Winter, Magdalena Meyer, Ilona Berger, Melanie Royer, Marta Bianchi, Kerstin Kuffner, Sebastian Peters, Simone Stang, Dominik Langgartner, Finn Hartmann, Anna K. Schmidtner, Stefan O. Reber, Oliver J. Bosch, Anna Bludau, David A. Slattery, Erwin H. van den Burg, Benjamin Jurek, Inga D. Neumann

**Affiliations:** 1 Department of Behavioural and Molecular Neurobiology, Regensburg Center of Neuroscience, University of Regensburg, Regensburg, Germany; 2 Department of Neurology, University Hospital Regensburg, Regensburg, Germany; 3 Laboratory for Molecular Psychosomatics, Department of Psychosomatic Medicine and Psychotherapy, University of Ulm, Ulm, Germany; 4 Laboratory of Translational Psychiatry, Department of Psychiatry, Psychosomatic Medicine and Psychotherapy, University of Frankfurt, Frankfurt am Main, Germany; 5 Center for Psychiatric Neurosciences, University Hospital Lausanne, Lausanne, Switzerland; 6 Institute for Molecular and Cellular Anatomy, University of Regensburg, Regensburg, Germany

**Keywords:** Cell biology, Neuroscience, Molecular biology, Biochemistry, Biological techniques

## Abstract

The neuropeptide oxytocin (OXT) has generated considerable interest as potential treatment for psychiatric disorders, including anxiety and autism spectrum disorders. However, the behavioral and molecular consequences associated with chronic OXT treatment and chronic receptor (OXTR) activation have scarcely been studied, despite the potential therapeutic long-term use of intranasal OXT. Here, we reveal that chronic OXT treatment over two weeks increased anxiety-like behavior in rats, with higher sensitivity in females, contrasting the well-known anxiolytic effect of acute OXT. The increase in anxiety was transient and waned 5 days after the infusion has ended. The behavioral effects of chronic OXT were paralleled by activation of an intracellular signaling pathway, which ultimately led to alternative splicing of hypothalamic corticotropin-releasing factor receptor 2α (*Crfr2α*), an important modulator of anxiety. In detail, chronic OXT shifted the splicing ratio from the anxiolytic membrane-bound (mCRFR2α) form of CRFR2α towards the soluble CRFR2α (sCRFR2α) form. Experimental induction of alternative splicing mimicked the anxiogenic effects of chronic OXT, while sCRFR2α-knock down reduced anxiety-related behavior of male rats. Furthermore, chronic OXT treatment triggered the release of sCRFR2α into the cerebrospinal fluid with sCRFR2α levels positively correlating with anxiety-like behavior. In summary, we revealed that the shifted splicing ratio towards expression of the anxiogenic sCRFR2α underlies the adverse effects of chronic OXT treatment on anxiety.

## Introduction

Neuropeptides and their receptors are established modulators of neuronal activity, shaping multiple behavioral and physiological responses to environmental stimuli. Neuropeptide signaling is, therefore, an important target for the development of pharmacological treatments of psychopathologies, such as autism-spectrum, anxiety and substance-use disorders, or schizophrenia, among others [1, 2] associated with social and emotional dysfunctions. In this context, the neuropeptide oxytocin (OXT) has received considerable attention over the last decades due to its profound prosocial, fear-reducing and anxiolytic effects demonstrated both in human and animal studies [3]. Studies in rodents have shown that the multiple behavioral effects of acute OXT can be induced within the paraventricular nucleus of the hypothalamus (PVN) [4, 5], central amygdala [6–8], lateral septum [9, 10], and cortical [11] and brainstem [12] regions.

OXT is synthesized in the PVN and supraoptic nuclei of the hypothalamus, where it is released somato-dendritically in response to stressful and fear-enhancing stimuli [3, 13, 14]. Such locally-released OXT, e.g., within the PVN, as well as acute local OXT infusions, exert robust anxiolytic effects in rats, shown in several relevant behavioral tests [4, 5]. Within the PVN, OXT also acts as neuromodulator of social behaviors [15, 16] and physiological functions including the regulation of the hypothalamic-pituitary-adrenal axis and stress-induced secretion of adrenocorticotropin (ACTH) and corticosterone [17, 18].

On a molecular level, the G protein-coupled OXT receptor (OXTR) is linked to multiple intraneuronal signaling cascades [3, 19]. In brief, the G protein α_q_ subunit is at the basis of activation of protein kinase C (PKC) [20], whereas the β/γ subunit activates Ca^2+^-influx through transient receptor potential vanilloid type 2 (TRPV2) and other Ca^2+^ channels [3, 21]. This is followed by the recruitment of mitogen-activated protein kinase (MAPK) kinase (MEK1/2) signaling [21] and protein synthesis [20]. Both MEK1/2 signaling and protein synthesis are necessary for the acute anxiolytic effect of OXT in the PVN of male [4, 20] and virgin female [5] rats. In addition, the acute anxiolytic activity of OXT in the PVN is enhanced under mild stress conditions [4] indicating an interaction of OXT and central stress regulation. Indeed, OXT has been found to reduce and delay the hypothalamic expression of corticotropin-releasing factor (*Crf*) [22, 23], and reciprocal interactions with the anxiolytic [24, 25] transmembrane CRF receptor 2α (mCRFR2α) signaling in the PVN and bed nucleus of the stria terminalis have been described [26, 27].

OXT was found to be behaviorally effective not only in laboratory animals [3, 28], but also in humans, where synthetic OXT can be applied intranasally [29, 30]. In most human studies, OXT has been applied acutely so far, and, even at relatively high doses, persistent side effects have not been reported [31, 32]. However, we and others have found adverse effects on anxiety [33], fear [34], and social behaviors [35–37] after chronic OXT application, or otherwise artificially enhanced OXT signaling, in rodents. For example, we have recently observed a dose-dependent anxiogenic effect in mice following continuous intracerebroventricular (icv) infusion of OXT using osmotic minipumps, with a dose of 10 ng/h OXT increasing anxiety-like behavior after 14 days [33]. These adverse behavioral consequences were accompanied by a reduction in intracerebral OXTR expression [33, 35]. In line with the described consequences of chronic OXT treatment and increased OXTR-mediated signaling, viral vector-induced overexpression of the OXTR in the lateral septum enhanced contextual fear in socially defeated mice [34]. Only in female rats with genetically determined high anxiety [38] and in lactating females [39], a beneficial anxiolytic effect of icv OXT infusion over 5 days was found. However, the molecular mechanisms underlying the adverse effects of chronic or prolonged OXT actions on anxiety, fear, or social behavior, in contrast to its beneficial acute effects, have mostly been disregarded, but ought to be carefully considered before OXT can be used as a treatment option for psychiatric disorders [28, 29, 40–42]. So far, the few human studies employing repeated intranasal administration of OXT over several days or weeks focused on social parameters and almost consistently reported a lack of major adverse side effects, such as self-reported anxiety or changes in blood pressure, osmotic homeostasis or bone metabolism [43, 44], but more detailed and targeted studies are missing.

Therefore, in the present study we addressed the molecular mechanisms causing the anxiogenic effect of chronic icv OXT treatment in rats. While we found that chronic OXT enhanced anxiety-like behavior in both male and female rats, the underlying mechanism appeared to be sexually dimorphic. Uniquely in males, chronic OXT recruited a so far unknown intracellular signaling pathway in the PVN downstream of the OXTR, which includes the MAPK-controlled phosphorylation of the transcription factor myocyte enhancer factor 2 isoform A (MEF2A) [45]. In more detail, all MEF2 isoforms are crucial transcription factors controlling basic cellular functions associated with learning and memory consolidation, dendrite morphogenesis, excitatory synapse formation in hippocampal neurons during development [46], and some variants are associated with autism spectrum disorder [47, 48]. The MEF2A-specific phospho sites Ser408, Thr312, Thr319 assessed in this study have different transcriptional effects, with Ser408 being repressive, and Thr312 and Thr319 being transcription activators. Further downstream of MEF2A, we could reveal that chronic icv OXT induced alternative splicing of *Crfr2α* to its soluble form (sCRFR2α). Interestingly, chronic OXT also triggered the release of the splice variant sCRFR2α into the cerebrospinal fluid (CSF), and we further found that sCRFR2α levels in CSF positively correlated with anxiety-like behavior. As a final proof of the involvement of sCRFR2α in the adverse behavioral consequences of chronic OXT treatment, we manipulated *Crfr2α* splicing in favor of its soluble form and could confirm its anxiogenic regulatory capacity. Taken together, our data provide a mechanistic explanation of the anxiogenic effect of chronic OXT in male rats, and illustrate the intimate relationship between the OXT and CRF systems. Consequently, they challenge the concept of treating emotional or social dysfunctions with repetitive or chronic OXT application [40, 49–51], and forward sCRFR2α as a novel target for the development of anxiolytics.

## Material and methods

A detailed list of chemicals and material used in this study can be found in Supplementary Table S1. Primers and antibodies are listed in Supplementary Tables S2 and S3.

### Animals and husbandry

Adult male and female Wistar rats (Charles River, Germany, 250–300 g) were housed under standard temperature- and humidity-controlled conditions with food and water *ad libitum*. All animal experiments were performed between 08:00–11:00 according to the ARRIVE guidelines [52] and recommendations from the National Institutes of Health, and approved by the government of Unterfranken, Germany. In all in vivo experiments, the experimenter was blind to the treatment. Group sizes were estimated upon power analysis, based on results from previous publications [5, 22, 33, 38]. Animals were randomly assigned to experimental groups, complying with equal mean body weight between groups. Pre-established exclusion criteria for animals included complications during surgery, poor histological quality, and general poor health condition.

### Chronic icv OXT infusion

Osmotic minipumps (Alzet, model 1002, flow rate 0.25 µl/h, 14 days) were filled with either vehicle (VEH; Ringer’s solution, B. Braun Melsungen AG, Germany), 4 µM or 40 µM OXT (Bachem, Bubendorf, Switzerland) to allow the infusion of 1 ng/h or 10 ng/h of OXT, and were implanted subcutaneously [38]. On day 13, rats were mildly stressed by 5-min exposure on the elevated platform [4, 53], and tested in the light-dark box (LDB) [15, 38] on day 14. Another rat cohort was tested in the LDB on day 14 without prior platform exposure.

### Acute icv and local intra-PVN infusions

Acute icv or intra-PVN infusions of VEH, OXT (icv: 100 ng/5 µl; local: 10 ng/0.5 µl per side, corresponding to 20 µM), antisauvagine-30 (ASV, 500 ng/0.5 µl per side; Tocris Bioscience, Bristol, UK) or stresscopin (SCP, 3 µg/0.5 µl per side, 25 min prior testing; Phoenix Pharmaceuticals, Inc., Burlingame, USA) were performed 7 days after stereotaxic implantation of guide cannulas targeting the region of interest and recovery as described before [4, 15, 53].

### Infusion of GapmeRs or target site blockers (TSBs)

In order to assess the behavioral consequences of selective manipulation of mCRFR2α/sCRFR2α ratio, bilateral intra-PVN (−1.7 mm bregma, ±0.3 mm lateral, 8.2 mm deep) infusions of locked nucleic acid antisense oligonucleotides (so-called GapmeRs), TSBs or scrambled control oligonucleotides (0.5nmol/0.5 µl, Qiagen, Hilden, Germany, Supplementary Material) were performed 7 days prior to LDB testing, and 8 days prior to testing in the open field (OF) and for social preference [54].

### Behavioral tests

Anxiety-related behavior was assessed in the LDB, elevated plus-maze (EPM), or OF during 5-min sessions [4]. For behavioral specificity of OXT effects in the PVN, social motivation was tested in the social preference test [54]. Locomotion (distance traveled) was determined by Noldus EthoVision XT 14 software.

### Tissue, plasma, and organ processing

After behavioral testing, brains were removed for regional protein and RNA isolation, histological verification of cannula placement or in situ hybridization (ISH). For CSF sampling from the cisterna magna, rats were terminally anaesthetized with urethane, before trunk blood was collected for analysis of ACTH and corticosterone levels. The weights of adrenal glands, heart, and thymus were taken, and oil-red staining of lipid vesicles in the adrenal cortex was conducted [55].

### Cell lines and primary cultures

Authentication of rat hypothalamic H32 cells [56] was executed on the basis of OXTR sequencing, morphology, and marker gene expression. Potential mycoplasma contamination was assessed on a regular basis. Cells were cultured in DMEM/F12 (Sigma-Aldrich, Darmstadt, Germany) with 10% heat inactivated FBS advanced (Capricorn, Ebsdorfergrund, Germany), and penicillin/streptomycin (Sigma-Aldrich, Darmstadt, Germany). Cells were sub-cultured by gentle trypsinization (Gibco™, Thermo Fisher Scientific, Waltham, USA) at 80% confluence. For transcriptional analyses, cells were seeded at a density of 3 × 10^6^ cells in a 25 cm² cell culture dish the day before experiment, pre-incubated in serum-free stimulation medium for 1 h and stimulated with the respective treatment.

Primary neuronal and glial cultures were obtained from embryonic day 18 rat hypothalami [22].

### In silico analysis of MEF2 targets

Analysis of potential MEF2 targets by assessment of DNA binding regions was performed using the Geneious prime software (Geneious prime 2019.0.3; https://www.geneious.com).

### MEF2A knockdown studies

H32 cells were seeded 24 h prior to transfections. For MEF2A knockdown, siRNA or scrambled RNA (scrRNA) as control (1 nM; #SR504191, OriGene, Rockville, USA, Supplementary Material) were transfected with Lipofectamine RNAiMAX (Invitrogen by Thermo Fisher Scientific, Waltham, USA) and incubated for 72 h at 37 °C and 5%CO_2_. After 48 h, cells were stimulated with either 100 nM OXT or VEH for another 24 h.

### Chromatin-IP

H32 cells were stimulated as described above, MEF2A-DNA complexes were fixed for 10 min, lysed (Supplementary Material), sheared by sonication, precleared with Sepharose beads, and MEF2A complexes isolated with a specific MEF2A antibody (Supplementary Material). DNA fragments were identified by qPCR with primers directed against *Crfr2*-specific MEF2A binding sequences (Supplementary Material).

### Nano-Glo® HiBiT extracellular detection system

The integration site of the HiBiT-containing Ultramer® ssDNA donor sequence was directly upstream of Exon 6 of the *Crfr2α* gene (Supplementary Material). The donor sequence was delivered together with the RNP complex (Alt-R® CRISPR-Cas9 system, IDT, Coralville, USA) by using Lipofectamine™ RNAiMAX. Membrane-bound CRFR2α (mCRFR2α) was visualized using the Nano-Glo® HiBiT Extracellular Detection System according to the manufacturer’s instructions (Promega, Mannheim, Germany). Luminescence was measured at the GloMax Explorer (Promega, Mannheim, Germany).

### Immunofluorescent labeling for co-localization of sCRFR2α, OXTR, and OXT in OXTR-reporter mice

OXTR-reporter mouse brains (OXTR-Venus [12]) were immunostained (Supplementary Material). Colocalization of sCRFR2α, OXTR and OXT was analyzed using the BioVoxxel Version of Fiji and plugin JACoP. Antibody specificity was assessed by pre-incubation with immunizing peptide, and in knockdown and overexpression systems (Supplementary Fig. S1A–D).

### Immunocytochemistry

To visualize sCRFR2α expression, immunocytochemistry in primary hypothalamic cells was performed (Supplementary Material).

### Western Blot and Dot Blot analysis

For Dot Blot analysis, 10 µg of total protein was pipetted onto a Nitrocellulose membrane, allowed to dry and processed identical to the Western Blot protocol previously described [20, 22, 45]. Detection was performed using a specific sCRFR2α antibody [57]. Loading was controlled by Ponceau red staining.

### ISH for OXT mRNA

To assess hypothalamic OXT mRNA, ISH was conducted [33] using a ^35^S-labeled probe specific for rats and mice (Supplementary Material).

### Receptor autoradiography for OXTR and V1aR binding

To quantify hypothalamic OXTR and V1aR binding, receptor autoradiography was performed on 16-µm coronal cryostat sections [39] (Supplementary Material).

### ACTH and corticosterone analyses

Plasma samples were assayed using ELISA (IBL, Hamburg, Germany) for ACTH (sensitivity 0.22 pg/ml, intra-assay and inter-assay coefficients of variation ≤ 7.1%) and corticosterone (sensitivity < 1.63nmol/l, intra-assay and inter-assay coefficients of variation ≤ 6.35%).

### RNA isolation for qPCR and PCR array

To analyze mRNA expression of target genes in punches from PVN, hippocampus, and prefrontal cortex, RNA was isolated as described before [22].

To isolate RNA from stimulated H32 cells, the medium was aspirated, cells were washed with PBS, and RNA was isolated according to manufacturer’s instruction (Macherey Nagel, Düren, Germany).

300 ng of total RNA per sample were used for reverse transcription into cDNA using Super Script IV First strand Synthesis System for RT-PCR (Invitrogen; Supplementary Material). The custom RT^2^ PCR array (330171 CLAR25389) was purchased from Qiagen (Hilden, Germany) and pipetted according to the manufacturer’s protocol.

### TransAM MEF2 binding kit

Protein samples were collected according to the manufacturer’s protocol (Active Motif, Rixensart, Belgium), and 20 µg protein was loaded onto the pre-coated 96 well plate. MEF2A and MEF2C subform-specific antibodies from OriGene/Acris (Rockville, USA; Supplementary Material) were used. Fluorescence was determined at 450 nm in a plate reader (FluoStar Optima, BMG LABTECH, Ortenberg, Germany).

### Statistical analysis

Parametric one-way (factor treatment) or two-way (factors treatment x time) analysis of variance (ANOVA), followed by Holm Sidak post hoc correction, were performed for statistical analyses of behavioral and molecular experiments (Sigma Plot, version 11.0.0.75, Systat Software). Data sets were analyzed for normal distribution using the Shapiro–Wilk test. Non-parametric data was analyzed by Kruskal–Wallis ANOVA on ranks and Tukey post hoc test. Separate parametric *t*-test between two groups or non-parametric Mann–Whitney U tests were performed. Data outliers were defined as above or below mean ± 2x standard deviation. Statistical significance was accepted at *p* < 0.05. Data are presented as mean ± standard error of the mean (SEM).

## Results

### Chronic OXT dose-dependently induces an anxiogenic phenotype

**Fig. 1:**
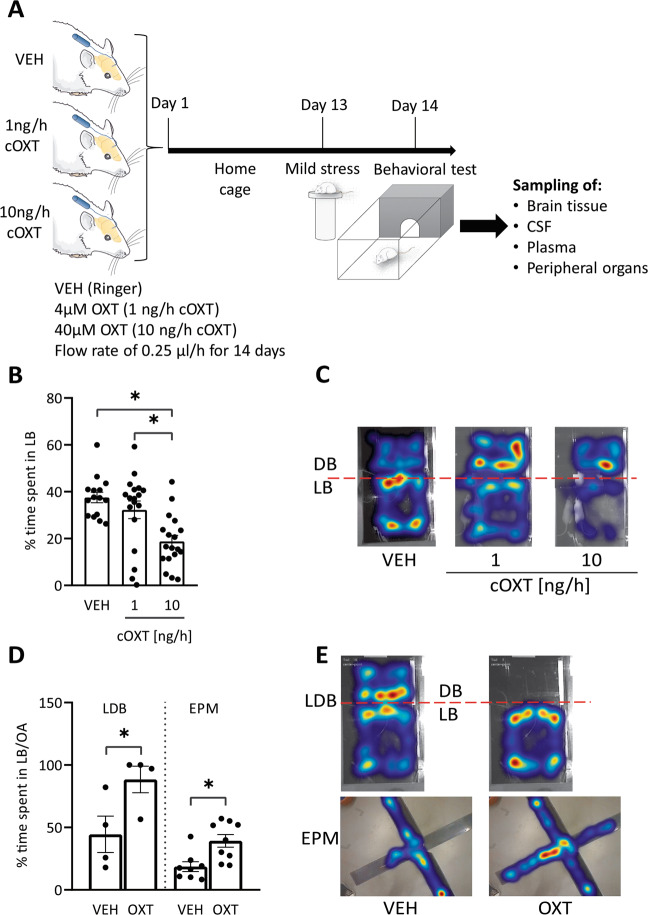
Chronic icv OXT infusion increases anxiety in male rats. **A** Schematic of the chronic OXT (cOXT) infusion paradigm (created with BioRender.com). Osmotic minipumps (flow rate 0.25 µl/h, infusion duration 14 days) were filled with vehicle (VEH), 4 or 40 µM OXT, corresponding to 1 and 10 ng/h of OXT release rates, respectively, subcutaneously implanted and connected to the lateral ventricle of rats via polyethylene tubing. Day 1 marks the start of infusions. After 13 days, rats were mildly stressed by exposure to the elevated platform (EPF) for 5 min, and tested in the light-dark box (LDB) on day 14. Brain samples were collected immediately after LDB exposure. **B** cOXT decreased the time spent in the light box in a dose-dependent manner. Shown is the percentage of time spent in the lit box (LB). Data are represented as mean ± SEM. F(2;50) = 10.131; *p* < 0.001; Holm-Sidak *p < 0.001 vs VEH; *n*(VEH) = 15, *n*(1 ng/h, 10 ng/h) = 18. **C** Representative heat maps of rat location in the LDB; treatments as described in **B**. **D** Acute bilateral infusion of OXT (acOXT; 20 µM, 0.01nmol/0.5 µl per side) into the paraventricular nucleus (PVN) reduced anxiety-related behavior. Male rats were tested in the LDB or elevated plus-maze (EPM) 10 min after infusion, and 24 h after EPF exposure. Shown is the percentage of time spent in the LB or open arm (OA). Data are represented as mean ± SEM. One-tailed Student’s *t* test, *p* < 0.01, df = 6; *n*(VEH) = 4, *n*(acOXT) = 4. **E** Representative heat maps of rat location in the LDB and EPM after intra-PVN VEH or acOXT treatment.

After 14 days of chronic icv OXT infusion, and one day after 5-min exposure to the elevated platform, rats were tested in the LDB (Fig. 1A). Compared to VEH controls, males treated with the high (10 ng/h), but not low (1 ng/h), dose of OXT spent less time in the light box, demonstrating an anxiogenic effect of chronic OXT (Fig. 1B, C). The increase in anxiety contrasted with the anxiolytic effect seen after acute OXT infusion into the PVN under otherwise identical experimental conditions (Fig. 1D, E), and with the absence of any effect on anxiety-like behavior after acute icv infusion (Supplementary Fig. S2A [38]). The anxiogenic phenotype was transient and waned within five days after termination of chronic OXT infusion (Supplementary Fig. S2B). In females, the lower dose increased anxiety-like behavior, indicating seemingly higher sensitivity, while the higher dose was without effect in the LDB (Supplementary Fig. S2C). Chronic OXT-induced anxiogenesis was not accompanied by changes in locomotor activity, neither in males nor in females (Supplementary Table S4). Importantly, chronic OXT had no effect on the expression of hypothalamic OXT and AVP, their receptors, or on OXTR and V1aR binding in various brain regions (Supplementary Table S5). Also, plasma ACTH and corticosterone levels, as well as other peripheral parameters remained unchanged after chronic OXT (Supplementary Tables S5 and S6 and Supplementary Fig. S2D).

### Chronic OXT treatment recruits the transcription factor MEF2A

**Fig. 2:**
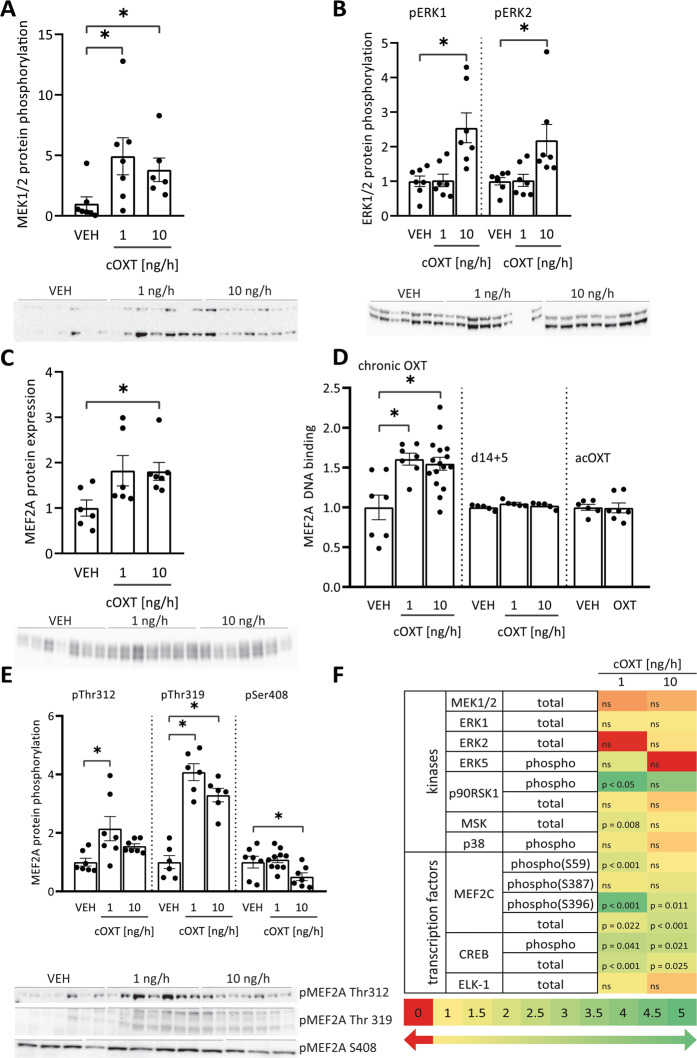
Recruitment of MAPK and MEF2A by chronic OXT infusion in the PVN of male rats. **A** cOXT at low and high doses increased MEK1/2 phosphorylation in the PVN after 14 days. Data are represented as mean ± SEM. Kruskal–Wallis *H* = 8.396; *p* = 0.015; Dunn’s Method *p < 0.05 vs VEH; *n*(VEH; 1 ng/h) = 7, *n*(10 ng/h) = 6. **B** ERK1/2 phosphorylation was increased by 10 ng/h cOXT treatment; *n*(VEH; 1 ng/h; 10 ng/h) = 7; pERK1: F(2;20) = 9.672; *p* = 0.001; Holm-Sidak *p = 0.001 VEH, 1 ng/h vs 10 ng/h; pERK2: Kruskal–Wallis *H* = 10.293 *p* = 0.006; Tukey Test VEH vs 10 ng/h *p < 0.05, 1 ng/h vs 10 ng/h *p < 0.05. **C** MEF2A total protein expression increased in PVN lysates of 10 ng/h cOXT-treated male rats. Kruskal–Wallis *H* = 7.156, *p* = 0.028; Dunn’s Method *p < 0.05 VEH versus 10 ng/h; *n*(VEH) = 6, *n*(1 ng/h) = 6, *n*(10 ng/h) = 7. **D** MEF2A DNA binding activity increased in PVN lysates after 14 days of chronic OXT at both doses and mild stress. Values are normalized to VEH. F(2;29) = 8.262, *p* = 0.002, Holm-Sidak *p = 0.05 vs VEH; *n*(VEH) = 7, *n*(1 ng/h) = 7, *n*(10 ng/h) = 16. In contrast to 14 days of cOXT treatment, MEF2A binding activity returned to basal, 5 days after the infusion had stopped (cOXT d14 + 5). Data are represented as fold changes in DNA-binding activity ±SEM. *F*(2,14) = 2.316, *p* = 0.141; *n*(VEH) = 5, *n*(1 ng cOXT) = 5, *n*(10 ng cOXT) = 5. No effects of acute intra-PVN OXT infusions have been observed on local MEF2A binding activity. *t* = 0.0938; two-tailed *p* value = 0,927, *n*(VEH) = 6, *n*(OXT) = 7; *n* for all groups *n* = 7. **E** Phosphorylation of Thr312 and Thr319 within MEF2A increased in both the high and the low treatment group, while Ser408 phosphorylation decreased after cOXT at 10 ng/h. pMEF2A Thr312: Kruskal–Wallis *H* = 9,106, *p* = 0.011; Tukey Test VEH vs 1 ng/h *p < 0.05; *n*(VEH) = 7, *n*(1 ng/h) = 7, *n*(10 ng/h) = 7. pMEF2A Thr319: *F*(2;17) = 41.139; *p* < 0.001; Holm-Sidak *p = 0.05 VEH versus 1 ng/h and 10 ng/h, and 1 ng/h versus 10 ng/h; *n*(VEH) = 6, *n*(1 ng/h) = 6, *n*(10 ng/h) = 6. pMEF2A Ser408: *F*(2;23) = 4.614; *p* = 0.022; Holm-Sidak *p = 0.033 VEH versus 10 ng/h cOXT; *n*(VEH) = 7, *n*(1 ng/h) = 10, *n*(10 ng/h) = 7. **F** Heat map of expression and phosphorylation levels of second messenger kinases and transcription factors downstream of the OXTR as analyzed by Western blot. Fold change below 1 (red) indicates downregulation, fold change of 1 (yellow) indicates no change, fold change bigger than 1 indicates upregulation (green). *p* values vs VEH included; n.s. not significant; *n* for all groups *n* = 7.

In PVN protein extracts, we observed increased phosphorylation, reflecting activation, of the MAP kinases MEK1/2 (at 1 and 10 ng/h) and ERK1/2 (at 10 ng/h) after chronic OXT infusion in males, whereas no such activation was found in females (Fig. 2A, B, Supplementary Fig. S3A, B). Moreover, chronic OXT (10 ng/h) resulted in elevated total protein levels of MEF2A (Fig. 2C), and this was accompanied by increased MEF2A activity, as indicated by increased MEF2A DNA binding in PVN tissue lysates of male rats (Fig. 2D, Supplementary Fig. S3C). The observed phosphorylation of the transcription activation sites Thr312 and Thr319 reflects elevated MEF2A activity, whereas the MEF2A inhibitory site Ser408 was dephosphorylated (Fig. 2E). Thus, chronic OXT activates MEF2A in the PVN of male rats (Supplementary Table S5 and Supplementary Fig. S3D). In contrast, acute icv OXT failed to enhance MEF2A activity (Supplementary Fig. S3E). Thus, OXTR-coupled signaling underlying the acute anxiolytic effect in the PVN does not involve the activation of MEF2A (Fig. 2D).

Five days after chronic OXT infusion had ended, MEF2A-DNA binding capacity was similar to that seen in VEH-treated rats (Fig. 2D), indicating that MEF2A activation is reversible. The activation of MEF2A in the PVN of chronically OXT-infused male rats appeared to be highly specific, as none of the other related kinases tested, including p38, p90rsk, and MSK, showed any significant changes in phosphorylation (Fig. 2F). In addition, the phosphorylation pattern of the closely related MEF2C, strongly expressed in the PVN and linked to OXTR signaling [58], remained unchanged at Ser59, the regulatory site inducing DNA binding (Fig. 2F). Consequently, DNA binding of MEF2C remained unaffected in the group treated with 10 ng/h OXT (Supplementary Fig. S3F).

In conclusion, chronic central infusion of high OXT results in MEF2A activation in the PVN of male rats, and increased anxiety-related behavior. However, recruitment of MEF2A does not underlie the anxiogenic effect induced by infusion of the low dose of OXT in virgin female rats. Thus, the intracellular mechanisms causing anxiogenesis in females are distinct from those in males, and need further investigation.

### MEF2A shifts the expression of membrane-bound to soluble CRFR2α in the PVN

**Fig. 3:**
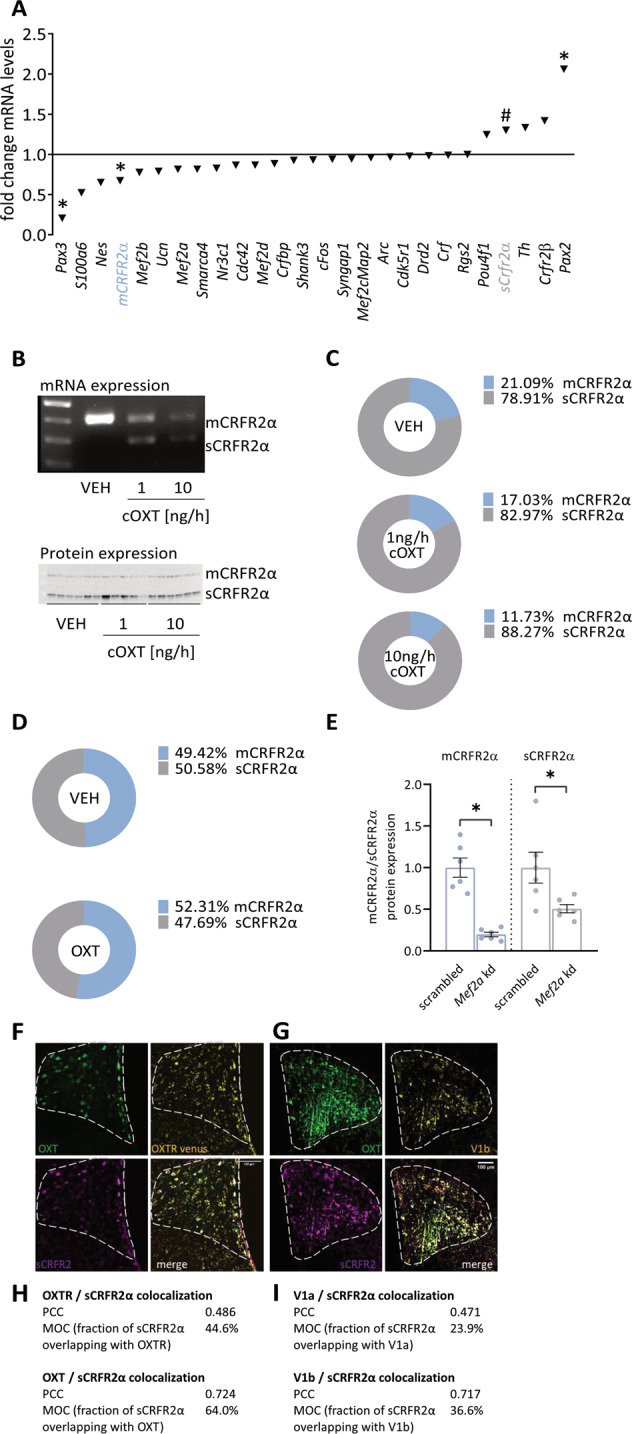
MEF2A-dependent regulation of OXT receptor target genes after cOXT treatment. **A** PCR array: Expression of MEF2A-regulated candidate genes after 10 ng/h cOXT treatment. Data calculated by ΔΔCT and presented as fold change vs. VEH. Fold changes > 1, upregulated mRNA expression, fold changes < 1, downregulated mRNA expression. *n*(VEH) = 5, *n*(10 ng/h cOXT) = 6. Pax3 *p = 0.021, *Crfr2α* *p = 0.02, *sCrfr2α* ^#^*p* = 0.07, Pax2 *p = 0.05. **B** Upper panel: Representative example of 3 male rat PVN cDNA samples showing *mCrfr2α* (upper band, 400 bp) in VEH-treated animals, and *mCrfr2α* / *sCrfr2α* (lower band, 300 bp) expression in the cOXT treatment groups. Lower panel: Western Blot of mCRFR2α (~50 kDa) and sCRFR2α (~30 kDa) levels after VEH, 1 or 10 ng/h cOXT infusion and mild stress, using a polyclonal pan-CRFR2 antibody. **C** Ratio of mCRFR2α and sCRFR2α protein expression after cOXT and mild stress in male rats, shifting in the favor of sCRFR2α following treatment. Data are represented as mean ± SEM. *F*(2;15) = 5.311, *p* = 0.021; Holm-Sidak *p* = 0.019 10 ng/h vs VEH; *n*(VEH) = 6, *n*(1 ng/h) = 5, *n*(10 ng/h) = 6. **D** Treatment with acOXT had no effects on the ratio of mCRFR2α/sCRFR2α protein expression in % in the PVN of male rats. Data are represented as mean ± SEM. *t* = 0.784 with 11 degrees of freedom, two-tailed *p* value = 0.450; *n*(VEH) = 6, *n*(OXT) = 7. **E** siRNA-mediated knockdown of MEF2A in H32 cells incubated with 100 nM OXT decreased mCRFR2α and sCRFR2α protein levels, indicating a central role for MEF2A in the transcription of the CRFR2 gene. Data represented as mean ± SEM. mCRFR2α: Mann–Whitney Rank Sum Test <0.001, *p = 0.002; sCRFR2α: *t* = 2.574 with 10 degrees of freedom; one-tailed *p value = 0.0138, both vs. respective scr (scrambled) control; *n*(scr) = 6, *n*(MEF2A kd) = 6. **F** Representative stainings showing co-localization of OXT-neurophysin I (green), sCRFR2α (magenta), and OXTR-Venus (yellow) in the PVN (indicated by white dotted line) of male OXTR-Venus reporter mice. **G** Representative staining showing colocalization of OXT (green), sCRFR2α (magenta), and V1bR (yellow) in the PVN of male Wistar rats. **H** Pearson and Manders overlap coefficient of OXTR, OXT, and sCRFR2α positive neurons reveal substantial (45–64%) overlap between the OXTR, OXT, and sCRFR2α. **I** Pearson and Manders coefficient reveal partial (24–37%) overlap of V1a/b and sCRFR2α in the PVN. OXT-neurophysin and sCRFR2α show similar co-expression (~60%) in rats and mice, indicating a comparable expression pattern of the sCRFR2α in both species.

To identify potential MEF2A target genes, we used a custom-designed PCR array that targets genes in the PVN that 1) are related to MEF2A functions, such as synaptic connectivity or neuronal plasticity, 2) contain one or more MEF2A binding sequences, and 3) have been associated with stress- or anxiety-like behaviors. After high chronic OXT treatment, we found distinct alterations in gene expression of closely related genes, of which those of membrane-bound and soluble CRFR2α were particularly striking (Fig. 3A), because of the known OXT and CRF interactions [22, 59], as well as the involvement of CRF in stress and anxiety [25, 60–62]. The expression of membrane-bound CRFR2α (mCRFR2α; Fig. 3A) was reduced, whereas that of the alternative splice variant, soluble CRFR2α (sCRFR2α), was increased in PVN tissue of chronic OXT-treated male, but not female (Supplementary Fig. S4A), rats, when compared with VEH-treated animals. Alternative splicing was confirmed by endpoint PCR demonstrating that *sCrfr2α* expression, which was below the detection limit in VEH controls, is effectively induced after chronic OXT (Fig. 3B upper panel). At the protein level, OXT-treated males expressed more sCRFR2α (88.3%) in proportion to mCRFR2α (11.7%; Fig. 3B lower panel and 3 C). No such effect was seen after an acute local administration of OXT (Fig. 3D). In silico analysis revealed that the coding sequence of the *Crfr2α* gene harbors seven potential MEF2A binding sites, two of them present within Exon 6 (base pairs 791–800 and 908–917). We performed ChIP analysis (Supplementary Fig. S4B) with H32 cells, which express MEF2A, OXTR (Supplementary Fig. S1A, B) and CRFR2α and found MEF2A binding to the *Crfr2α* gene especially within Exons 2 and 6 (Supplementary Fig. S4B). Furthermore, selective MEF2A knockdown by means of siRNA strongly reduced both mCRFR2α and sCRFR2α expression in H32 cells, highlighting the necessity of MEF2A to induce *Crfr2α* gene transcription (via binding to Exon 2) and to promote alternative splicing (via binding to Exon 6; Fig. 3E). In confirmation of the presence of the OXTR-MEF2A-sCRFR2α cascade within OXTR-expressing PVN neurons, we found that OXTR (as revealed by a Venus signal) and sCRFR2α are partially co-expressed in the PVN of OXTR reporter mice [12], whereas almost no co-expression of sCRFR2α with V1aR or V1bR was detectable (Fig. 3F–I).

### Chronic OXT promotes the release of sCRFR2α in vitro

**Fig. 4:**
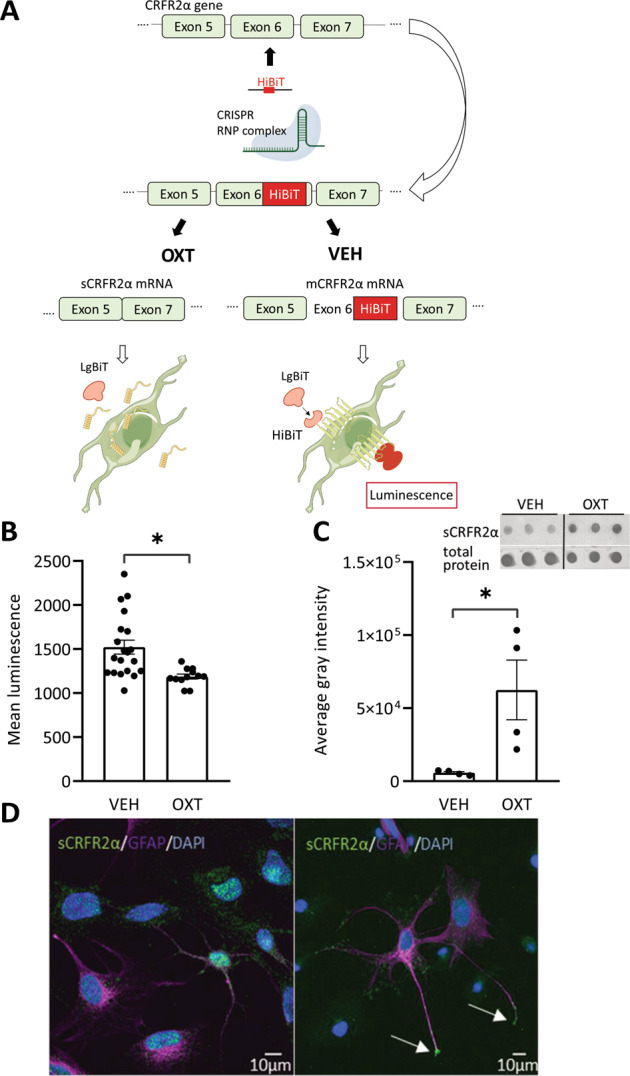
Chronic OXT promotes the release of sCRFR2α in vitro. **A** Schematic representation of HiBiT-mediated signaling in H32 cells stimulated with VEH or OXT for 24 h (created with BioRender.com). **B** Assessment of mCRFR2α membrane expression by tagging the transmembrane domain encoded in Exon 6 with a HiBiT-tag. Membrane expression is indicated by luminescence caused by extracellular HiBiT-LgBiT interaction. Stimulation for 24 h with 100 nM OXT decreased the luminescent signal, indicating alternative splicing of Exon 6 and, therefore, reduced membrane expression of CRFR2α. Data presented as absolute values of mean luminescence ±SEM. Mann–Whitney Rank Sum Test, *p ≤ 0.001, *n*(VEH) = 20, *n*(OXT) = 12. **C** Cell culture supernatants taken from HiBiT-expressing cells (as shown in **B**) reveal sCRFR2α release into the cell culture medium, which was 10-fold higher after stimulation with 100 nM OXT over 24 h. Data shown as average gray intensity relative to total protein Ponceau red staining. Mann–Whitney Rank Sum Test, *p = 0.029; *n*(VEH/OXT) = 4. Right panel: Representative Dot Blot of triplet sCRFR2α staining and Ponceau red loading control. **D** Left panel: Representative images from rat hypothalamic mixed primary cultures stained for DAPI (blue), sCRFR2α (green), and GFAP (magenta) reveal cytoplasmic distribution of sCRFR2a in neuronal cells (GFAP negative) and astrocytes (GFAP positive). Right panel: Accumulation of sCRFR2α in end-boutons of astrocytic processes (white arrows) might indicate releasable pools.

To identify and visualize mCRFR2α in H32 cells, we integrated a spliceable tag (HiBiT) into Exon 6 of *Crfr2α* by means of CRISPR-Cas9. This tag was visualized by adding the LgBiT substrate to the cell culture medium (Fig. 4A). The luminescent signal that forms following binding of LgBiT and HiBiT detects mCRFR2α in live cells. We found that luminescence intensity dropped in OXT-treated cells (100 nM, 24 h), indicating reduced membrane incorporation of mCRFR2α (Fig. 4B). In parallel, sCRFR2α content increased tenfold in the cell culture medium (Fig. 4C) demonstrating OXT-induced release of sCRFR2α from H32 cells. In addition, staining of primary hypothalamic cells revealed punctate sCRFR2α immunoreactivity in neurons and glial cells, reflecting vesicular localization (Fig. 4D).

### Changes in mCRFR2α/sCRFR2α ratio result in increased anxiety

**Fig. 5:**
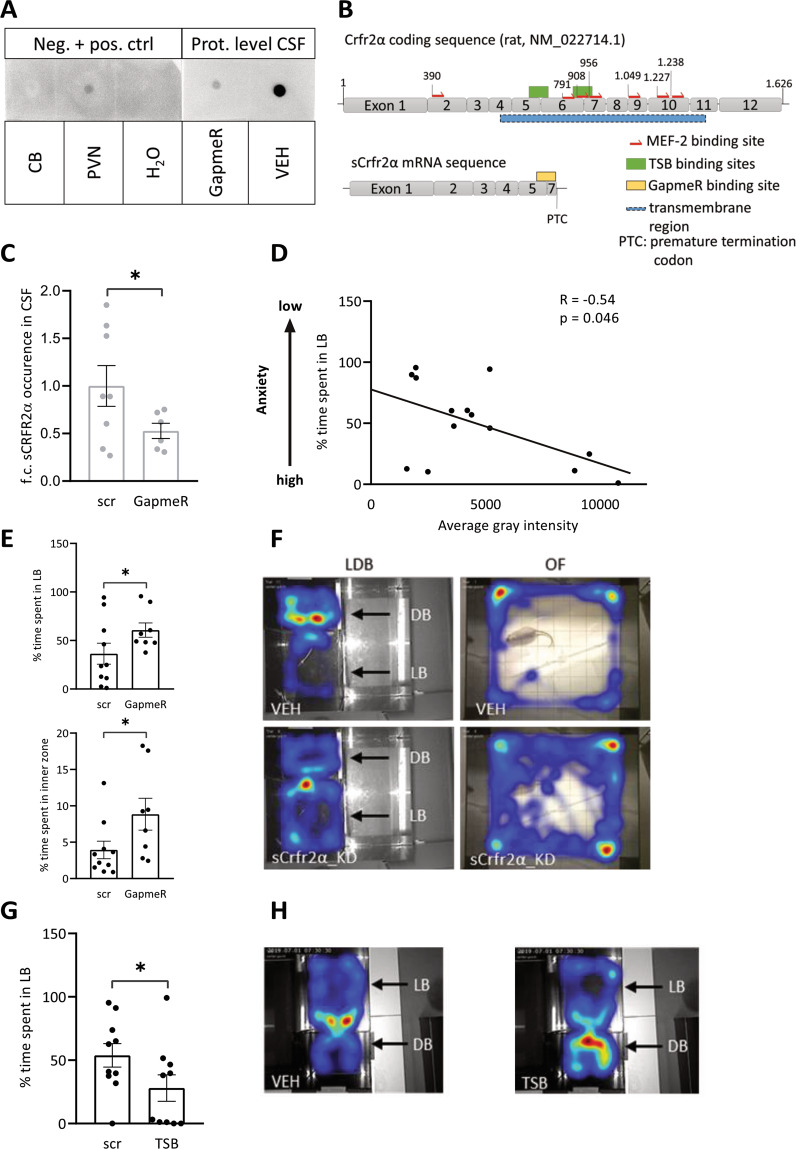
Increased sCRFR2α release underlies the anxiogenic effect of chronic OXT in combination with mild stress. **A** Representative picture of Dot Blot analysis (for detailed methodology see Supplementary Methods) of CSF (VEH and GapmeR) and control tissue protein lysates (cerebellum = CB as negative control, PVN as positive control, H_2_O). For detection, the sCRFR2α-specific antibody (provided by Joan Vaughan [57]) was used at a dilution of 1:20.000 in 5% BSA. **B** Schematic representation of the rat *Crfr2α* gene, the transmembrane domain is indicated by a blue bar, including Exon 4 to Exon 11. Target site blocker (TSB) binding sites are indicated by green bars. Multiple MEF2 binding sites are indicated as red arrows. Exon 5/7 boundary GapmeR binding site in the *sCrfr2α* mRNA is indicated in yellow. **C** Infusion of GapmeRs targeting *sCrfr2α* bilaterally into the PVN of male rats reduced sCRFR2α expression by ~50% as assessed by Dot Blot in CSF samples. Data are shown as fold change in protein expression ±SEM. One-tailed Student’s *t* test, *t* = 1.828, df = 12, *p = 0.046; *n*(scr) = 8, *n*(GapmeR) = 6. **D** Individual CSF sCRFR2α signal intensity and % time spent in LB correlate negatively (*R* = −0.54, ANOVA *F*(1;13) = 4.946, *p* = 0.046), suggesting that sCRFR2α is anxiogenic. **E** GapmeR-induced reduction in sCRFR2α increased the time spent in LB and inner zone of the OF, indicating anxiolysis. Anxiety-like behavior was determined 7 (LDB) or 8 days (OF) after GapmeR infusion. Data are represented as mean percentage of time spent in the LB ± SEM (one-tailed *t*-test *p = 0.0486), and the mean percentage of time in the inner zone of the OF (Mann–Whitney Rank Sum Test *U* = 16.000, *p = 0.037) ±SEM; *n*(scr) = 10, *n*(GapmeR) = 8. **F** Representative heat maps showing the distribution of rat presence in the light and dark compartment of the LDB or inner and outer zone in the OF test. **G** Infusion of TSBs bilaterally into the PVN of male rats and local induction of alternative splicing of *Crfr2α* enhanced anxiety-like behavior of male rats in the LDB 7 days after infusion, demonstrating an anxiogenic effect of sCRFR2α. Data are represented as mean percentage of time spent in the LB ± SEM. *t* = 1.843; one-tailed *t*-test *p = 0.0409; *n*(scr) = 10, *n*(TSB) = 10. **H** Representative heat map of the distribution of rat presence in the LDB after VEH or TSB treatment.

To determine whether sCRFR2α is released in vivo, we sampled CSF from the cisterna magna of male rats and found detectable amounts of sCRFR2α (Fig. 5A). In contrast, sCRFR2α was not detectable in cerebellum tissue [57], confirming the specificity of the dot blot analysis. Next, we specifically knocked down the sCRFR2α splice variant in the PVN by local infusion of GapmeRs [63] and assessed whether the diminished availability of sCRFR2α affects anxiety-related behavior. The GapmeRs were designed to bind the Exon 5–7 boundary, which is exclusively found in the mRNA of the soluble form of CRFR2α, so that specific knockdown of sCRFR2α was achieved (Fig. 5B). Fluorescent labeling of the infused GapmeRs revealed predominantly intra-PVN staining, with some diffuse signal scattered in the adjacent, mostly dorsal hypothalamus, potentially representing cellular projections originating from the PVN (Supplementary Fig. S5A). Infusion of GapmeRs into the PVN caused a significant reduction of sCRFR2α in the CSF after 7 days (Fig. 5C), which was accompanied by a reduction in anxiety-like behavior in the LDB reflected by an increased time spent in the light box and increased exploration of the center area of the light box (Fig. 5D–F). In a complementary approach, we upregulated sCRFR2α levels by TSBs, to promote alternative splicing of the *Crfr2α* mRNA to its shorter *sCrfr2α* form. This was achieved by targeting TSBs to the endogenous splice sites up- and downstream of Exon 6, thus enforcing the exclusion of Exon 6 from the pre-mRNA and inducing a frame shift to form a premature termination codon within Exon 7 (Fig. 5B). This process results in predominant *sCrfr2α* mRNA levels, at the expense of reduced *mCrfr2α* levels. TSB infusion bilaterally into the PVN resulted in increased anxiety-related behavior of male rats in the LDB (Fig. 5G, H).

Taken together, these results indicate that a low mCRFR2α/high sCRFR2α ratio in the PVN is associated with an anxiogenic phenotype. This was further substantiated by our observation of anxiolytic properties of mCRFR2α, as activation of mCRFR2α by the agonist (SCP), and blocking mCRFR2α with the antagonist (ASV) produced anxiolytic and anxiogenic effects, respectively (Supplementary Fig. S5B). Of note, the consequences of altered mCRFR2α/sCRFR2α ratio appear to be specific for anxiety, as social preference behavior, known to be promoted by OXT [9, 54], was not affected by GapmeRs or TSBs (Supplementary Fig. S5C). In summary, the identified OXTR-ERK1/2-MEF2A-sCRFR2α intraneuronal pathway may underlie the anxiogenic effect of chronic OXT treatment in male rats.

## Discussion

The brain OXT system has repeatedly been suggested as target for the treatment of psychopathologies associated with symptoms of anxiety [29, 40, 64]. Given that synthetic OXT therapy would most likely involve chronic or repeated application, detailed knowledge of the behavioral and molecular processes downstream of the OXTR after chronic OXT is indispensable.

In this study, we revealed that chronic icv OXT for 14 days increases anxiety-like behavior in male and virgin female rats in a sex- and dose-dependent manner. This central effect seems to be of transient nature, as it disappeared after a 5-day washout period. This together with the lack of an acute icv OXT effect on anxiety-like behavior indicates a reversible molecular mechanism within the brain that underlies the anxiogenic effect of chronic OXT. We showed that the anxiogenic effect in males is accompanied by the activation of a novel intracellular signaling pathway involving increased transcriptional activity of MEF2A, and subsequent alternative splicing of *Crfr2α* into its soluble, anxiogenic, form. The distinct role of sCRFR2α in anxiety regulation was confirmed by targeted manipulations of *Crfr2α* splicing. While knockdown of sCRFR2α within the PVN using GapmeRs reduced anxiety, local upregulation of alternative splicing of *Crfr2α* using TSBs increased anxiety, thus recapitulating the effect of chronic OXT. We further showed by CRISPR-Cas9-guided insertion of a HiBiT-tag into Exon 6 of the *Crfr2α* gene that chronic OXT reduced membrane expression of mCRFR2α and induced the release of sCRFR2α in vitro. In vivo, sCRFR2α levels could be detected and quantified in the CSF, and were found to positively correlate with anxiety-like behavior. Although our results challenge the utility of chronic or repetitive OXT application as treatment option for anxiety-related disorders, we could identify sCRFR2α as a novel target for diagnosis and pharmacological intervention of these disorders.

The CRFR2α is predominantly expressed in the brain, with a few exceptions in peripheral organs [65, 66]. One characteristic of the shorter and soluble splice variant, sCRFR2α, is its inability to incorporate into the cellular membrane due to the lack of the transmembrane domain and, therefore, high abundance in the cytosol from where it can be released into the extracellular space [57, 67]. Previous publications have suggested that the process of alternative splicing serves as a mechanism to control full-length GPCR mRNA availability, which is in line with our data revealing the shifted ratio between sCRFR2α/mCRFR2α after chronic OXT treatment [67, 68].

We found that OXT-induced changes in MEF2A activity are involved in alternative splicing of *Crfr2α*, in line with recent findings that MEF2A is part of a large complex of transcription and splicing-regulating factors, such as Brg1/Brm, BAF47, BAF170, BAF155, or MyoD. These factors contribute to the correct transcription and immediate processing of the transcript [69, 70]. Thus, our results support the hypothesis that transcription and alternative splicing are linked and co-dependent processes. Our data further suggest that activation of MEF2A via dephosphorylation at Ser408 is crucial to drive alternative splicing of *Crfr2α*, as this occurred only in the male 10 ng/h group and parallel to the increase in anxiety. Interestingly, chronic OXT did not activate MEF2A in female rats, although females appeared to be more sensitive to the anxiogenic effects of chronic OXT treatment than males. As a result, the mCRFR2α/sCRFR2α ratio remained unaltered in females after chronic OXT. Sexual dimorphism in OXT-induced anxiety has been described before and appears to be rooted in differential CRF signaling from OXTR-expressing interneurons in the prefrontal cortex [59]. In more detail, OXT-induced recruitment of CRF binding protein in the prefrontal cortex acts anxiolytic in a sex-specific manner, as CRF binding protein attenuates potentiation of postsynaptic layer 2/3 pyramidal cell activity only in male mice and, thereby, brings about anxiolysis [59]. Although shorter in length, sCRFR2α maintains its ability to bind the mCRFR2α and CRFR1 ligands CRF, urocortin I and urocortin II with nanomolar affinity [57], and thus could prevent them from binding to mCRFR2α [24]. Consequently, scavenging mCRFR2α ligands by sCRFR2α could weaken or prevent the anxiolytic signal conveyed by mCRFR2α, and promote anxiety-like behavior in males. However, in females, chronic OXT-induced anxiety appears to be mediated independent of MEF2A activation and sCRFR2α production.

Importantly, we have detected sCRFR2α in rat brain CSF and cell culture medium, proving that the release of sCRFR2α into the extracellular space can be induced by OXT. The observed correlation between CSF sCRFR2α levels and anxiety-like behavior further supports our hypothesis that the balance between soluble and membrane-bound CRFR2α in the PVN in favor of the anxiogenic soluble splice variant is instrumental in promoting anxiety in male rats. Interestingly, we observed that brief exposure to a mild stressor, i.e., the elevated platform 24 h prior to testing, is required to induce anxiogenesis after chronic OXT treatment. As mild stress promotes the incorporation of anxiolytic mCRFR2α into the membrane [71–73], chronic OXT might interfere with this process by shifting the mCRFR2α/sCRFR2α ratio.

In conclusion, our study shows that chronic OXT dose-dependently enhances anxiety-like behavior in males and females, which involves the activation of the transcription factor MEF2A and subsequent alternative splicing of *Crfr2α* mRNA into the anxiogenic soluble form in male rats. Consequently, these findings together with previous data on adverse effects of chronic or repetitive OXT treatment in rodents and humans [33–37, 51] need to be carefully considered, before using chronic or repetitive administration of OXT for the treatment of psychopathologies. Our findings suggest that targeting sCRFR2α synthesis and release may open a more promising avenue for future treatment options, particularly in men.

### Supplementary information

Figure S1

Figure S2

Figure S3

Figure S4

Figure S5

Supplementary information
