# Supplementary figures and images for: Chronic oxytocin-driven alternative splicing of Crfr2α induces anxiety
Source: Mol Psychiatry. 2021 May 25;28(11):4742–55. doi: 10.1038/s41380-021-01141-x (PMC10914602; doi:10.1038/s41380-021-01141-x)

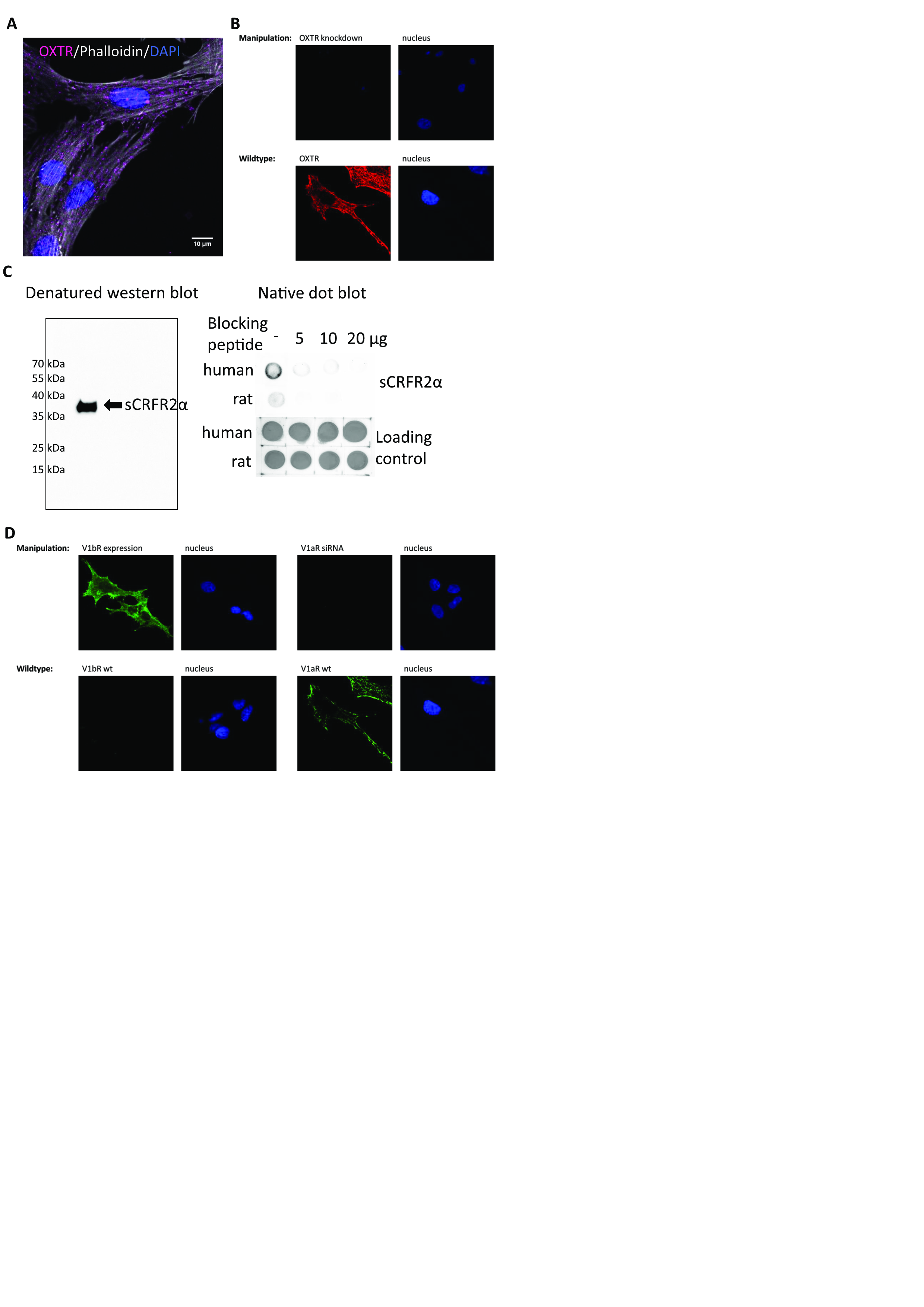

Supplement: Supplementary file 1 — Figure S1 [file 41380_2021_1141_MOESM1_ESM.tif]

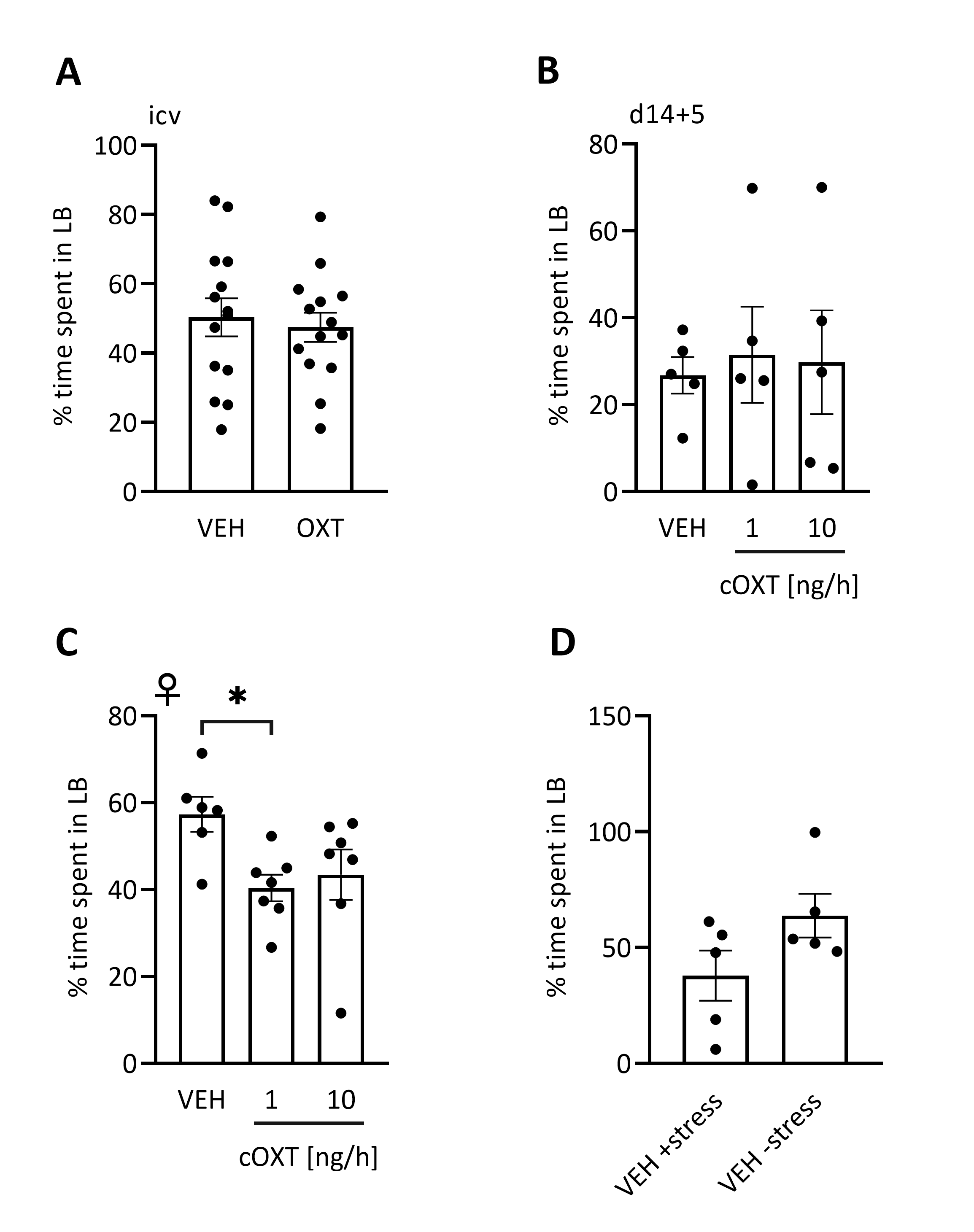

Supplement: Supplementary file 2 — Figure S2 [file 41380_2021_1141_MOESM2_ESM.tif]

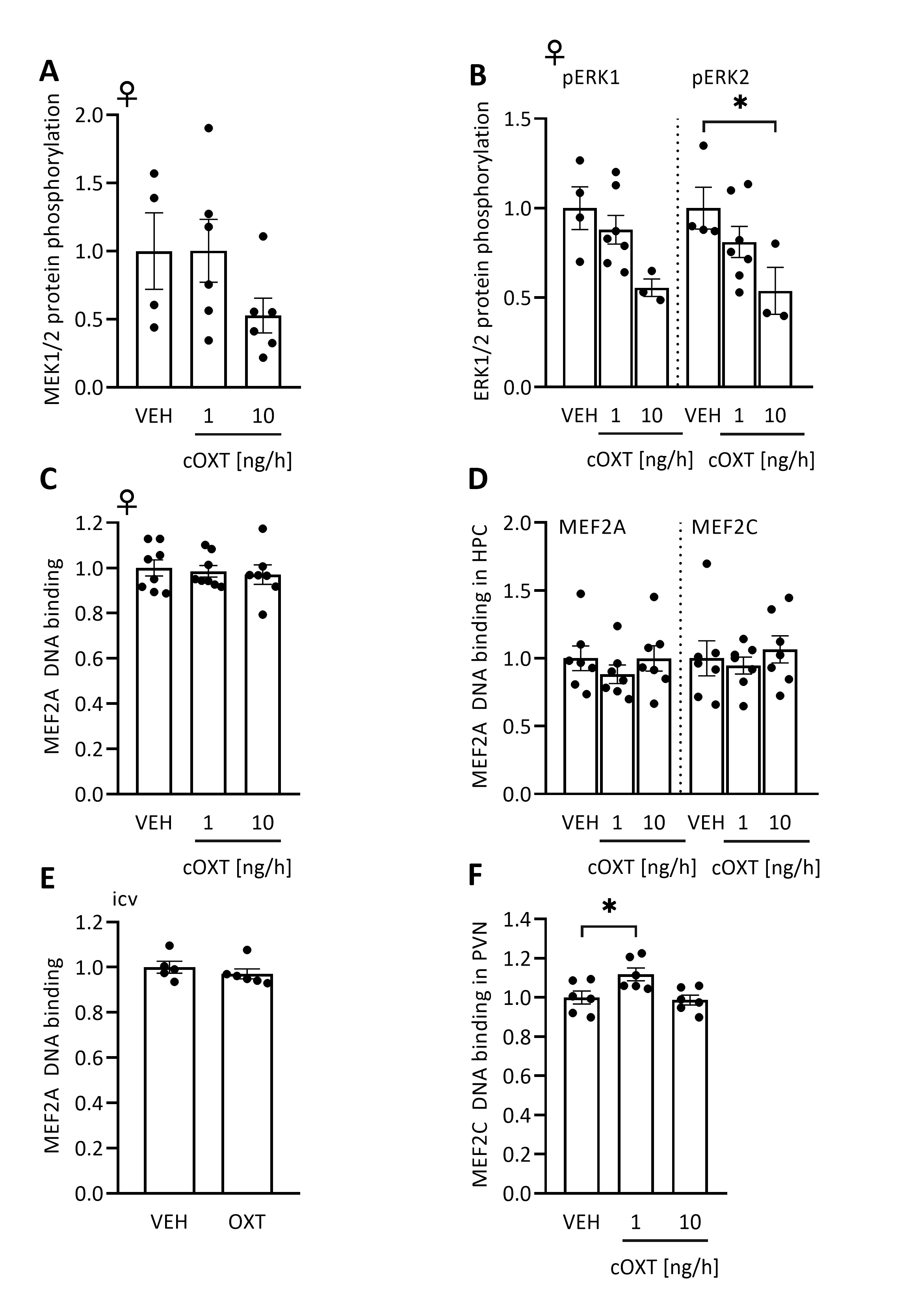

Supplement: Supplementary file 3 — Figure S3 [file 41380_2021_1141_MOESM3_ESM.tif]

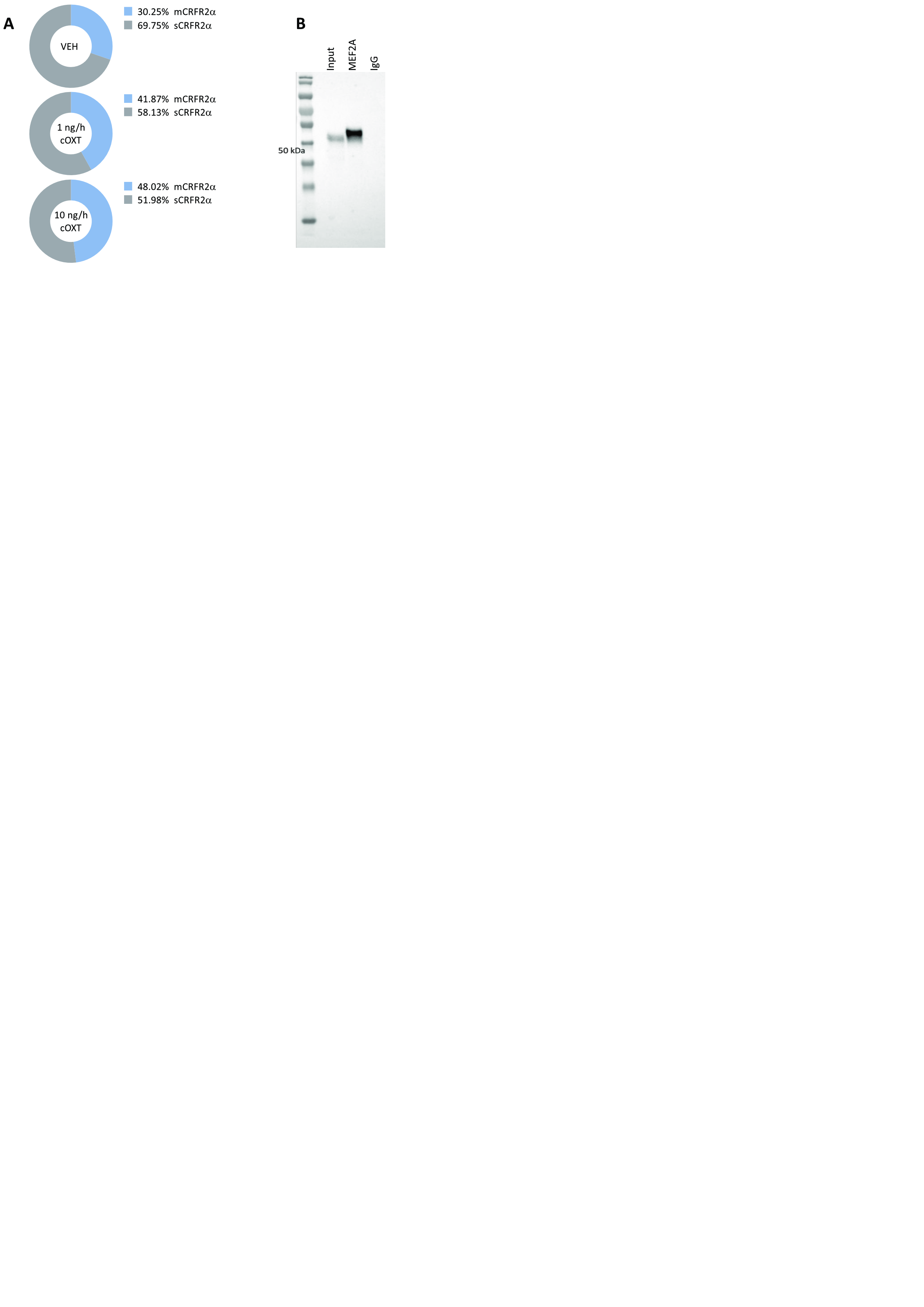

Supplement: Supplementary file 4 — Figure S4 [file 41380_2021_1141_MOESM4_ESM.tif]

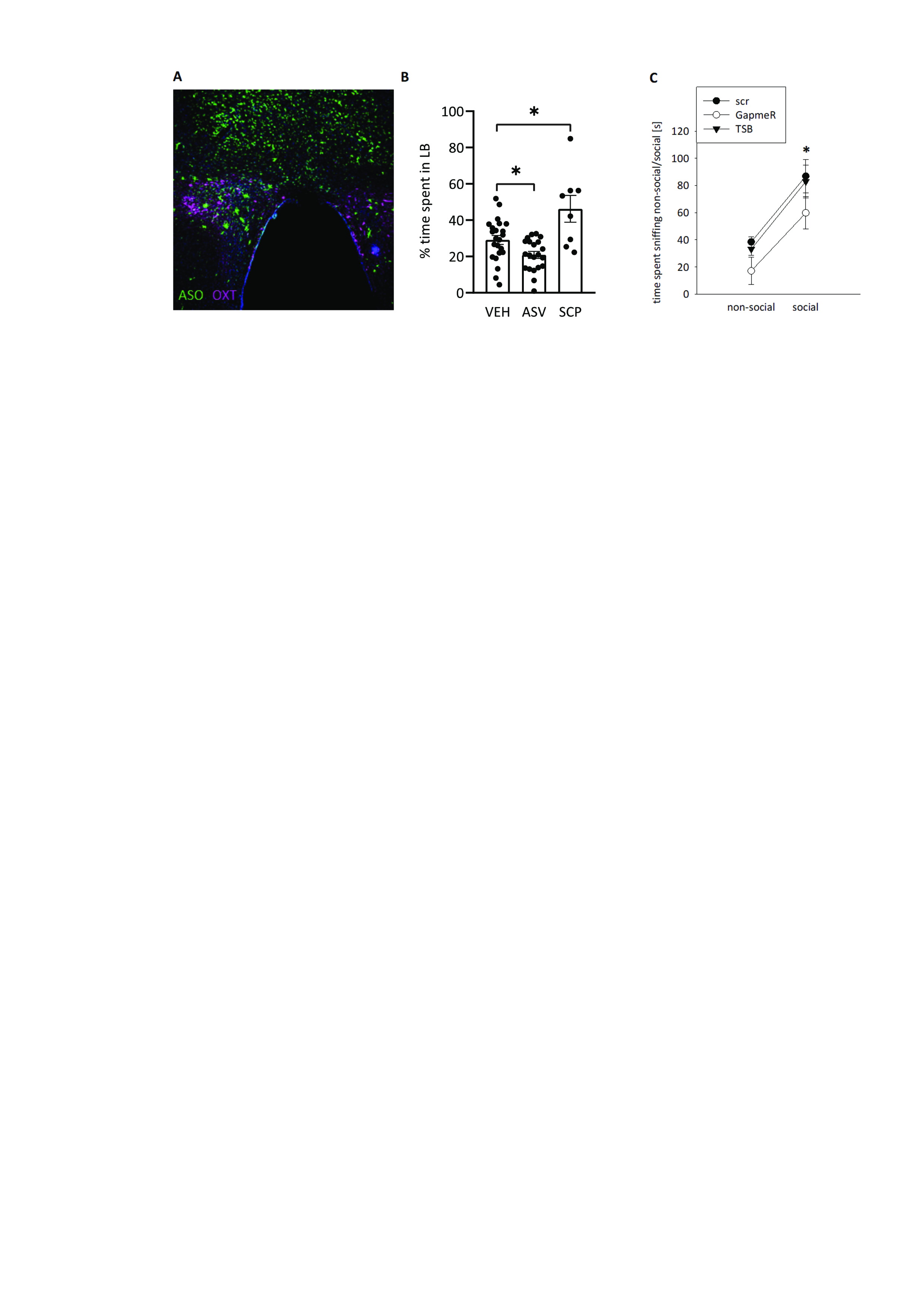

Supplement: Supplementary file 5 — Figure S5 [file 41380_2021_1141_MOESM5_ESM.tif]
