# Supplementary material for: Chronic oxytocin-driven alternative splicing of Crfr2α induces anxiety
Source: Mol Psychiatry. 2021 May 25;28(11):4742–55. doi: 10.1038/s41380-021-01141-x (PMC10914602; doi:10.1038/s41380-021-01141-x)
Supplement: Supplementary file 6 — Supplementary information [file 41380_2021_1141_MOESM6_ESM.docx]

**SUPPLEMENTARY INFORMATION (SI)**

**1. Material and Methods**

| **Chemicals, Peptides, and Recombinant Proteins** | | |
| --- | --- | --- |
| Baytril (Enrofloxacin) | Bayer, Leverkusen, Germany | Cat #09089728 |
| Buprenovet (Buprenorphin) | Bayer, Leverkusen, Germany | Cat #H018 |
| Oxytocin acetate salt | Bachem, Bubendorf, Switzerland | Cat #4016373.0005 |
| Antisauvagine-30 trifluoroacetate salt | Sigma Aldrich, Darmstadt, Germany | Cat #A4727 |
| Stresscopin | Abbiotec, Escondido, USA | Cat #350392 |
| Methylbutane | Sigma Aldrich, Darmstadt, Germany | Cat #M32631 |
| Urethane | Sigma Aldrich, Darmstadt, Germany | Cat #U2500 |
| Foetal bovine serum albumine | Capricorn Scientific, Ebsdorfergrund, Germany | Cat #FBS-HI-11A |
| Ethanol | Sigma Aldrich, Darmstadt, Germany | Cat #32205 |
| Trypsin-EDTA | Sigma Aldrich, Darmstadt, Germany | Cat #T4049 |
| Gentamycin | Sigma Aldrich, Darmstadt, Germany | Cat #G1397 |
| Glyoxal | Sigma Aldrich, Darmstadt, Germany | Cat #128465 |
| RNAiMax | Invitrogen by Thermo Fisher Scientific, Waltham, USA | Cat #13778 |
| DMEM/F12 | Sigma Aldrich, Darmstadt, Germany | Cat #D8437 |
| Poly-L-Lysine | Sigma Aldrich, Darmstadt, Germany | Cat #P8920 |
| ProLong Glass Anti-Fade Reagent with NucStain | Thermo Fisher Scientific, Waltham, USA | Cat #P36984 |
| TritonX | Sigma Aldrich, Darmstadt, Germany | Cat #T8787 |
| Bovine Serum Albumine | Sigma Aldrich, Darmstadt, Germany | Cat #A7906 |
| Glycine | Labochem international | Cat #LC-4522.2 |
| TriFast Gold | PeqLab, Radnor, USA | Cat #30-2010 |
| SybrGreen Power Up | Applied Biosystems, Foster City, USA | Cat #A25742 |
| Critical Commercial Assays | | |
| TransAM MEF2 binding Kit | Active Motif, Carlsbad, USA | Cat #43196 |
| Pierce BCA assay | Thermo Fisher Scientific, Waltham, USA | Cat #23227 |
| ACTH ELISA | IBL, Hamburg, Germany | Cat #RE53081 |
| CORT ELISA | IBL, Hamburg, Germany | Cat #RE52211 |
| Protein/RNA isolation Kit | Macherey Nagel, Düren, Germany | Cat #740933 |
| Custom RT² PCR array | Qiagen, Hilden, Germany | Cat #330171 |
| Nano-Glo HiBiT Extracellular detection system | Promega, Mannheim, Germany | Cat #N2420 |
| Alt-R CRISPR-Cas9 system | IDT, Coralville, USA | n.a. |
| **Experimental Models: Cell Lines** | | |
| H32 | provided by G. Aguilera, NICHD; created by J. Spiess | n.a. |
| H32^CRFR2-tg-HiBiT^ | created by J.Winter and B. Jurek | n.a. |
| **Experimental Models: Organisms/Strains** | | |
| Wistar rats | Charles River, Sulzfeld, Germany | n.a. |
| **Software and Algorithms** | | |
| Geneious 11.0.5 | Biomatters Ltd., Auckland, New Zealand | n.a. |
| Noldus EthoVision XT14 | Noldus, Wageningen, Netherlands | n.a. |
| Fiji ImageJ 1.52r | NIH, Bethesda, USA | n.a. |
| Sigma Plot 13.0 | Systat | n.a. |

**Table S1. Material list.**

**Post-surgery procedure**

Animals were randomly assigned to experimental groups, complying with equal mean body weight between groups. Following surgery, the animals received a subcutaneous injection of antibiotics (0.03 ml enrofloxacin; 100 mg/1ml) and Buprenorphine as analgesic (0.05 mg/kg). Rats that received an acute infusion underwent surgery as described in the material and methods section, single-housed, and allowed to recover for at least 5 days. Animals were handled daily to habituate them to the infusion procedure in order to avoid non-specific stress responses during the experiment.

**Antisense Oligonucleotides sequences**

GapmeR: 5’-FAM-*T*A*C*T*T*C*C*T*C*T*G*C*T*T*G-3’

TSB: 5’-FAM-*T*T*C*C*T*C*T*G*C*T*G*G*A*C*A-3’

5’-FAM-*T*G*G*A*C*T*C*A*C*C*G*C*A*G*C*A*C-3’

Scr: 5’-FAM-*A*C*G*T*C*T*A*T*A*C*G*C*C*C*A-3’

**ChIP**

Fixation Buffer:

11% Formaldehyd, 0.5 M HEPES, 0.1 M NaCl, 1 mM EDTA, 0.5 mM EGTA

MEF2A binding sequence-specific primers (Metabion, Munich, Germany):

XM_006236558.2

MEF binding sequence within Exon 2 at Pos. 390:

Fwd: TGGAGCCCTAGTGGAGAGAC

Rev: CCGGGTCGTGTTGTACTTGA

MEF binding sequence within Exon 6 at Pos. 791:

Fwd: GCTGCGTCACCACCATATTC

Rev: CAGGTAGCAGCCTTCCACAA

**HiBiT sequences**

gRNA: 5’-ACTTCCTCTGCTGGACAGAC-3’

HiBiT-encoding sequence: 5’-GCCCTACTCCTACATAGCCATGCCTCT-3’

Linker sequence: Ggatcatcaggaggatcatcagga

**Immunostaining protocol**

OXTR-reporter mouse brains were kindly provided by Prof. Nishimori. For immunostaining, 40 µm thick cryo cut sections containing the PVN were permeabilized, blocked (PBS with 2 % BSA, 1 % glycine, and 0.3 % TritonX-100, 1 h at room temperature), double labelled for sCRFR2α (rabbit polyclonal, provided by J. Vaughan, Salk Institute), in combination with OXT-Neurophysin I (mouse monoclonal, provided by Harold Gainer) and Venus-OXTR or V1a/b (V1aR: abbexa, Cambridge, UK, abx431978; V1bR: antibodies-online, Aachen, Germany, abin625884). The sections were gently transferred to SUPERFROST® glass slides and mounted with Surgipath Premier coverslips using ProLong Glass Anti-Fade Reagent with NucStain (# P36984, Thermo Fisher Scientific, Waltham, USA). Images were acquired using a SP8 CLSM confocal microscope (Leica, Wetzlar, Germany).

**ICC protocol** (primary cells)

0.05 x 10^6^ primary hypothalamic cells were seeded in BD Falcon Chamber slides (Poly-L-Lysine coated), fixated in 3 % glyoxal, rinsed in PBS-T, and blocked with blocking solution (PBS, 2 % BSA, and 0.5 % Triton X-100, 1 % glycine, 0.5 % cold water fish gelatine) for one hour followed by primary antibody (GFAP mouse monoclonal, Sigma Aldrich, Darmstadt, Germany, G3893) incubations in PBS-T (0.1 % Triton X-100) overnight and secondary Alexa Fluor antibodies for 2 h in PBS-T.

**Western Blot and Dot Blot analysis**

For Western Blot analysis, 25 µg of whole cell extract were loaded and separated in a 10 % Mini PROTEAN or Criterion TGX Stainfree gel (BioRad, Munich, Germany). Total protein and phosphorylated forms of various proteins (see antibody table S6), using the Stainfree total protein method (BioRad, Munich, Germany) as loading control.

For Dot Blot analysis, 10µg of total protein was pipetted onto a Nitrocellulose membrane, allowed to dry and processed identical to the Western Blot protocol. Loading was controlled by Ponceau red staining.

**Radiolabeled *in situ* hybridization probe**

The sequence for ^35^S-labeled probe: 5’CTC GGA GAA GGC AGA CTC AGG GTC GCA GGC GGG GTC GGT GCG GCA GCC 3’ is specific for rats and mice. Briefly, 16µm cryo-sections were mounted onto pre-coated SuperFrost Plus Slides (Menzel-Gläser, Braunschweig) and processed as described previously^1^. Brain slices, which contained comparable sections of PVN, were measured for each subject to provide individual means. Expression of OXT mRNA was measured as grey density with ImageJ 1.51 g. Background activity was automatically subtracted from measured areas to yield values for specific binding.

**Receptor autoradiography**

Rats were decapitated, brains were removed, quickly frozen in pre-chilled n-methylbutane on dry ice, and stored at -20 °C. Brains were cut into 16-mm coronal cryostat sections and mounted on slides. The using a linear V1aR antagonist ^125^I-phenylacetyl-d-Tyr(Me)-Phe-Gln-Asn-Arg-Pro-Arg-Tyr-NH2 (Perkin Elmer, Waltham, USA) or a linear OXTR antagonist [^125^I]-d(CH_2_)^5^[Tyr(Me)^2^-Tyr-Nh_2_]^9^-OVT (Perkin Elmer, Waltham, USA) as tracers. The optical density of V1aR and OXTR was measured using ImageJ (V1.51g). Receptor density was calculated per rat by taking the mean of bilateral measurements of four to six brain sections per region of interest. After subtraction of tissue background, the data was converted to dpm/mg (desintegrated points per minute/milligram tissue) using a [125I] standard microscale (Amersham, Germany).

**ACTH and CORT ELISA**

Approximately 1 ml of trunk blood was collected in EDTA-coated tubes on ice (Sarstedt, Nümbrecht, Germany) and centrifuged at 4 °C (5000 rpm, 10 min). Supernatant was removed and stored at -80°C as plasma samples, until assayed using a commercially available ELISA for ACTH (analytical sensitivity 0.22 pg/ml, intra-assay and interassay coefficients of variation ≤ 7.1 %, IBL, Hamburg) and CORT (sensitivity <1.63 nmol/l, intra-assay and inter-assay coefficients of variation ≤ 6.35 % IBL, Hamburg) and a 96 well plate reader (Optima FluoStar, BMG)

**Reverse transcription and qPCR**

300 ng of total RNA per sample were used for reverse transcription into cDNA using Super Script IV First strand Synthesis System for RT-PCR (Invitrogen by Thermo Fisher Scientific, Waltham, USA). Relative quantification of mRNA levels was performed using SYBR Green (QuantiFast Qiagen, Hilden, Germany), using ribosomal protein L13A as housekeeping gene^2^. Primer efficiency for each primer pair was calculated by serial dilution of test cDNA using the Pfaffl method^3, 4^.

| MEF2A (NM_001014035.1) | Metabion, Munich, Germany | Fwd: AAT GGG GCG AAA GAA AAT AC  Rev: GCT GGC GTA CTG AAA CAA CT |
| --- | --- | --- |
| sCRFR2α | Metabion, Munich, Germany | Fwd: CCC ATT TTG GAT GAC AAG GAG TA  Rev: GGA TGA AGG TGG TGA TGA GGT T |
| Rpl13A (NR_073024) | Metabion, Munich, Germany | Fwd: ACA AGA AAA AGC GGA TGG TG  Rev: TTC CGG TAA TGG ATC TTT GC |

**Table S2. Primer sequences.**

| pMEF2A Ser408 rabbit polyclonal | CusAbio, Wuhan, China | Cat #CSB-PA000728 |
| --- | --- | --- |
| pMEF2A Thr312 rabbit polyclonal | Abcam, Cambridge, UK | Cat #ab30644 |
| pMEF2A Thr319 rabbit polyclonal | OriGene, Rockville, USA | Cat #TA325686 |
| pMEF2C S396 rabbit polyclonal | OriGene, Rockville, USA | Cat #TA326044 |
| pMEF2C S387 mouse monoclonal | Santa Cruz Biotechnology, Dallas, USA | Cat #sc-377535 |
| pMEF2C S59 rabbit polyclonal | Santa Cruz Biotechnology, Dallas, USA | Cat #sc-13919-R |
| MEF2A total rabbit polyclonal | OriGene/Acris, Rockville, USA | Cat #AP06372PU-N |
| MEF2C total rabbit polyclonal | OriGene, Rockville, USA | Cat #TA326189, |
| MEF2A total mouse monoclonal | Santa Cruz Biotechnology, Dallas, USA | Cat #sc-17785 |
| CRFR2 rabbit polyclonal | Millipore, Burlington, USA | Cat #ABN 433 |
| pMEK1/2 (41G9) rabbit monoclonal | Cell Signaling, Leiden, Netherlands | Cat #9154 |
| MEK1/2 rabbit polyclonal | Cell Signaling, Leiden, Netherlands | Cat #9122 |
| CREB | Millipore, Burlington, USA | Cat #04-218 |
| pCREB S133 | Millipore, Burlington, USA | Cat #06-519 |
| Elk-1 | Cell Signaling, Leiden, Netherlands | Cat #9182 |
| pMSK1 | Abcam, Cambridge, UK | Cat #ab81294 |
| ERK1/2 | Cell Signaling, Leiden, Netherlands | Cat #9102 |
| pERK1/2 | Cell Signaling, Leiden, Netherlands | Cat #9101 |
| pERK5 | Millipore, Burlington, USA | Cat #07-507 |
| p90rsk1 | Santa Cruz Biotechnology, Dallas, USA | Cat #sc-231, |
| pp90rsk1 | Cell Signaling, Leiden, Netherlands | Cat #9344P |
| sCRFR2α | Provided by J. Vaughan, Salk Institute | n.a. |
| OXT-assoc. Neurophysin I antibody | Provided by Harold Gainer | PS38 |
| GFAP mouse monoclonal | Sigma Aldrich, Darmstadt, Germany | Cat #G3893 |
| Vasopressin Receptor V1a | Abbexa, Cambridge, UK | Cat #abx431978 |
| Vasopressin Receptor V1b | antibodies-online, Aachen, Germany | Cat #abin625884 |

**Table S3. Antibody details.**

**2. Data**

|  | **VEH** | **1ng/h cOXT** | **10ng/h cOXT** |
| --- | --- | --- | --- |
| **cOXT** | 1575.39 ± 83.80 | 1630.44 ± 93.20 | 1730.14 ± 264.43 |
| **cOXT females** | 2017.03 ± 154.96 | 1744.81 ± 87.02 | 2018.99 ± 191.96 |
|  | **VEH** | **acOXT** |  |
| **acOXT** | 1442.58 ± 76.79 | 1558.33 ± 63.95 |  |
|  | **VEH** | **ASV** | **SCP** |
| **ASV + SCP** | 2136.24 ± 125.95 | 2301.21 ± 110.50 | 2019.82 ± 107.54 |
| **GapmeR** | **VEH** | **GapmeR** |  |
| Open field | 2378 ± 326.79 | 3301.39 ± 167.42 |  |
| Light Dark Box | 1840.06 ± 147.39 | 2596.39 ± 155.98 |  |

**Table S4. Locomotion assessed in behavioral tests in [cm] by Noldus EthoVision** (cOXT: chronic oxytocin, acOXT: acute oxytocin, ASV: antisauvagine-30, SCP: stresscopin)**.**

| **Technique** | **Target** | **Brain region** | **Chronic OXT treatment 14 d male rats** | | | | **Significance vs Veh** | |
| --- | --- | --- | --- | --- | --- | --- | --- | --- |
|  |  |  | **VEH** | **1ng/h** | **10ng/h** |  | |  |
| *In situ* hybridisation | OXT mRNA | PVN | 86.64 ± 3.814 | 81.61 ± 8.51 | 86.95 ± 7.33 | n.s. | |  |
|  |  | SON | 70.68 ± 6.95 | 60.67 ± 8.32 | 70.20 ± 7.45 | n.s. | |  |
| Receptor autoradiography | OXTR binding | PVN | 24.04 ± 1.71 | 19.45 ± 1.76 | 19.74 ± 2.85 | n.s. | |  |
|  |  | SON | 18.03 ± 2.62 | 11.20 ± 0.98 | 14.59 ± 1.43 | n.s. | |  |
|  |  | DL Septum | 27.71 ± 2.54 | 25.33 ± 2.38 | 16.66 ± 2.36 * | *: p=0.04 | |  |
|  |  | Dorsal Raphe Nucleus | 22.38 ± 3.46 | 19.29 ± 1.54 | 19.40 ± 3.02 | n.s. | |  |
|  |  | Median Raphe Nucleus | 9.99 ± 1.33 | 7.44 ± 1.14 | 9.24 ± 1.29 | n.s. | |  |
|  |  | Nucleus Accumbens | 58.65 ± 6.5 | 50.41 ±5.6 | 71.77 ± 5.54 | n.s. | |  |
|  |  | CA1 | 16.83 ± 1.95 | 15.41 ±2.1 | 14.44 ± 1.32 | n.s. | |  |
|  |  | CA2 | 14.62 ± 1.68 | 14.34 ±1.64 | 13.85 ± 1.31 | n.s. | |  |
|  |  | CA3 | 12.93 ± 1.52 | 13.45 ± 1.15 | 10.72 ± 1.12 | n.s. | |  |
|  |  | Dentate Gyrus | 6.42 ± 0.98 | 6.53 ± 0.45 | 4.74 ± 0.44 | n.s. | |  |
|  |  | Central Amygdala | 111.12 ± 5.59 | 120.54 ± 1.5 | 114.33 ± 4.27 | n.s. | |  |
|  |  | Basolateral Amygdala | 32.52 ± 3.26 | 34.97 ± 1.77 | 30.25 ± 1.66 | n.s. | |  |
|  |  | Medial Amygdala | 39.22 ± 2.18 | 43.66 ± 2.08 | 38.56 ± 1.21 | n.s. | |  |
|  | AVPR binding | PVN | 15.98 ±2.13 | 18.007 ± 2.18 | 17.414 ± 1.24 | n.s. | |  |
|  |  | SON | 21.85 ± 1.828 | 23.036 ± 3.376 | 24.467 ± 1.364 | n.s. | |  |
|  |  | DL Septum | 31.70 ± 2.86 | 33.89 ± 2.70 | 37.71 ± 2.79 | n.s. | |  |
|  |  | Dorsal Raphe Nucleus | 46.59 ± 5.82 | 45.88 ± 2.95 | 45.5 ± 1.98 | n.s. | |  |
|  |  | Median Raphe Nucleus | 38.26 ± 3.49 | 36.73 ± 1.95 | 38.78 ± 2.28 | n.s. | |  |
|  |  | Nucleus accumbens | 22.96 ± 2.31 | 27.23 ± 1.75 | 24.97 ± 3.19 | n.s. | |  |
|  |  | CA1 | 23.40 ± 1.79 | 23.03 ± 0.36 | 22.82 ± 2.31 | n.s. | |  |
|  |  | CA2 | 21.96 ± 1.60 | 21.98 ± 0.72 | 21.39 ± 0.93 | n.s. | |  |
|  |  | CA3 | 16.45 ± 1.19 | 16.26 ± 0.94 | 16.99 ± 0.53 | n.s. | |  |
|  |  | Dentate Gyrus | 23.18 ± 3.76 | 24.08 ± 2.12 | 26.31 ± 3.04 | n.s. | |  |
|  |  | Central Amygdala | 48.63 ± 4.09 | 52.81 ± 1.76 | 56.76 ± 2.52 | n.s. | |  |
|  |  | Basolateral Amygdala | 25.73 ± 1.95 | 24.21 ± 1.09 | 25.57 ± 1.81 | n.s. | |  |
|  |  | Medial Amygdala | 23.09 ± 1.38 | 19.11 ± 1.25 | 20.95 ± 1.79 | n.s. | |  |
| qPCR | OXTR mRNA (fold change) | PVN | 1 ± 0.165 | 0.978 ± 0.141 | 0.950 ± 0.159 | n.s. | |  |
|  |  | Hippocampus | 1 ± 0.0076 | 0.996 ± 0.0063 | 1.01 ± 0.010 | n.s. | |  |
|  |  | Prefrontal Cortex | 1 ± 0.022 | 1.033 ± 0.006 | 1.0133 ± 0.0098 | n.s. | |  |
|  |  | Amygdala | 1 ± 0.1342 | 0.952 ± 0.181 | 1.157 ± 0.107 | n.s. | |  |
|  |  | Raphe Nucleus | 1 ± 0.190 | 1.349 ± 0.219 | 1.26 ± 0.226 | n.s. | |  |
|  |  | Septum | 1 ± 0.120 | 1.014 ± 0.184 | 1.083 ± 0.141 | n.s. | |  |
|  | V1a mRNA (fold change) | PVN | 1 ± 0.107 | 1.038 ± 0.108 | 1.34 ± 0.141 | n.s. | |  |
|  |  | Hippocampus | 1 ± 0.0066 | 0.985 ± 0.008 | 0.996 ± 0.0089 | n.s. | |  |
|  |  | Prefrontal Cortex | 1 ± 0.0094 | 0.999 ± 0.0038 | 0.986 ± 0.006 | n.s. | |  |
|  |  | Amygdala | 1 ± 0.0793 | 1.004 ± 0.072 | 1.086 ± 0.0795 | n.s. | |  |
|  |  | Raphe Nucleus | 1 ± 0.113 | 1.107 ± 0.118 | 1.023 ± 0.187 | n.s. | |  |
|  |  | Septum | 1 ± 0.132 | 0.86 ± 0.044 | 1.11 ± 0.0979 | n.s. | |  |
|  | MEF2A mRNA (fold change) | PVN | 1 ± 0.14 | 1.91 ± 0.38 * | 1.20 ± 0.09 | *: p=0.027 | |  |
|  |  | Hippocampus | 1 ± 0.0054 | 0.998 ± 0.0039 | 0.987 ± 0.0034 | n.s. | |  |
|  |  | Prefrontal Cortex | 1 ± 0.0049 | 0.99 ± 0.0046 | 1.013 ± 0.01 | n.s. | |  |
|  |  | Amygdala | 1 ± 0.004 | 1.00 ± 0.004 | 0.99 ± 0.005 | n.s. | |  |
|  |  | Raphe Nuclei | 1 ± 0.003 | 1.00 ± 0.003 | 1.00 ± 0.005 | n.s. | |  |
|  |  | Septum | 1 ± 0.003 | 1,003 ± 0.004 | 0,998 ± 0.002 | n.s. | |  |
|  | MEF2B mRNA (fold change) | PVN | 1 ± 0.16 | 0.89 ± 0.22 | 1.09 ± 0.12 | n.s. | |  |
|  |  | Hippocampus | 1 ± 0.0097 | 0.986 ± 0.0121 | 1.015 ± 0.0042 | n.s. | |  |
|  |  | Prefrontal Cortex | 1 ± 0.0047 | 1.008 ± 0.0033 | 0.996 ± 0.0048 | n.s. | |  |
|  | MEF2C mRNA (fold change) | PVN | 1 ± 0.18 | 0.56 ± 0.09 | 0.46 ± 0.07 * | *: p=0.047 | |  |
|  |  | Hippocampus | 1 ± 0.0096 | 0.988 ± 0.0134 | 1.005 ± 0.010 | n.s. | |  |
|  |  | Prefrontal Cortex | 1 ± 0.0052 | 1.0049 ± 0.0028 | 1.0598 ± 0.0188 | n.s. | |  |
|  |  | Amygdala | 1 ± 0.012 | 1.008 ± 0.006 | 1.017 ± 0.008 | n.s. | |  |
|  |  | Raphe Nuclei | 1 ± 0.03 | 1.010 ± 0.003 | 1.010 ± 0.008 | n.s. | |  |
|  |  | Septum | 1 ± 0.003 | 0.998 ± 0.014 | 1.004 ± 0.006 | n.s. | |  |
|  | CRF mRNA (fold change) | PVN | 1 ± 0.3002 | 1.01 ± 0.332 | 1.40 ± 0.281 | n.s. | |  |
|  | OXT mRNA (fold change) | PVN | 1 ± 0.183 | 0.879 ± 0.170 | 0.693 ± 0.191 | n.s. | |  |

**Table S5. Central effects of chronic OXT on gene or protein expression in stress- and anxiety-related brain regions evaluated by *in situ* hybridization, receptor autoradiography, or qPCR**. N.s. = not significant, n = 6-10 male rats, depending on the technique and experiment.

|  | **VEH** | **1ng/h chronic OXT** | **10ng/h chronic OXT** |
| --- | --- | --- | --- |
| plasma ACTH [pg/ml] | 53.056 ± 11.510 | 64.67 ± 14.41 | 50.03 ± 11.69 |
| plasma Cort with EPF but no LDB [ng/ml] | 389.29 ± 45.03 | 393.11 ± 58.06 | 463.53 ± 46.92 |
| Plasma Cort with EPF and with LDB [ng/ml] | 935.48 ± 59.84 | 638.53 ± 131.44 | 716.11 ± 139.65 |
| Plasma Cort no EPF but with LDB [ng/ml] | 839.54 ± 67.76 | 594.01 ± 74.00 | 805.99 ± 89.63 |
| rel. adrenal weight | 0.192 ± 0.015 | 0.179 ± 0.012 | 0.199 ± 0.014 |
| [mg/g body weight] |  |  |  |
| absolute adrenal weight left [g] | 0.036 ± 0.003 | 0.0354 ± 0.0013 | 0.0377 ± 0.0022 |
| absolute adrenal weight right [g] | 0.0342 ± 0.0022 | 0.0305 ± 0.0017 | 0.0352 ± 0.0016 |
| adrenal cortex lipid droplet content (oilred staining) [%] | 8.9 ± 0.01 | 8.9 ± 0.01 | 6.85 ± 0.01 |
|  |  |  |  |
| heart weight [g] | 0.9608 ± 0.0155 | 1.0965 ± 0.0401 | 1.0818 ± 0.0151 |
| body weight gain [g] | 14.4 ± 1.5 | 16.8 ± 0.80 | 16.3 ± 0.42 |
| rel. thymus weight | 1.38 ± 0.10 | 1.22 ± 0.06 | 1.32 ± 0.081 |
| [mg/g] |  |  |  |
| Dermal OXTR expression (fold-change mRNA) | 1.000 ± 0.03 | 0.952 ± 0.015 | 0.923 ± 0.038 |

**Table S6. Influence of chronic OXT treatment on peripheral parameters. None of the parameters assessed differed significantly between treatment groups.**

References

1. Peters S, Slattery DA, Uschold-Schmidt N, Reber SO, Neumann ID. Dose-dependent effects of chronic central infusion of oxytocin on anxiety, oxytocin receptor binding and stress-related parameters in mice. *Psychoneuroendocrinology* 2014; **42:** 225-236.

2. Bonefeld BE, Elfving B, Wegener G. Reference genes for normalization: a study of rat brain tissue. *Synapse* 2008; **62**(4)**:** 302-309.

3. Bustin SA, Benes V, Garson JA, Hellemans J, Huggett J, Kubista M *et al.* The MIQE guidelines: minimum information for publication of quantitative real-time PCR experiments. *Clinical chemistry* 2009; **55**(4)**:** 611-622.

4. Pfaffl MW. A new mathematical model for relative quantification in real-time RT-PCR. *Nucleic acids research* 2001; **29**(9)**:** e45.

**Figure S1.** **Antibody specificity and OXTR expression in H32 cells and *in vivo* co-localization analyses.**

(A) OXT receptor (OXTR) expression in H32 cells, OXTR in magenta, actin in white, DAPI in blue. Specificity of the Anti-OXT Receptor Antibody (AVR-013) from Alomone labs (Jerusalem, Israel) was tested and published elsewhere ^75^. (B) OXTR staining in H32 cells treated with OXTR siRNA (OriGene Oxtr rat siRNA Oligo Duplex (Locus ID 25342)) in comparison to H32 wildtype cells. (C) To confirm specificity of the sCRFR2α antibody, we tested it in a Western Blot using rat plasma under standard denaturing conditions (SDS, 95°C for 5 min). The antibody produced one single band at around 38 kDa, which is the predicted molecular weight. The right panel shows a dot blot with native rat plasma samples and increasing amounts of blocking peptide. Ponceau red staining prior to antibody labeling served as loading control, and confirms even protein loading onto the membrane. (D) left: V1bR staining in H32 cells treated with a V1bR expression plasmid (OriGene, Avpr1b (Myc-DDK-tagged ORF) in comparison to H32 wildtype cells with no endogenous V1bR expression. Right: V1aR staining in H32 cells treated with V1aR siRNA (OriGene, Avpr1b (Rat) - 3 unique 27mer siRNA duplexes) in comparison to endogenous expression in wildtype cells. Scale bar represents 10 µm.

**Figure S2. Continuative behavioral data.**

(A) Anxiety-like behavior of male rats in the LDB 10 min after icv OXT infusion at a dose of 100ng/5µL. Data are represented as mean % time spent in the LB ±SEM. t = 0.420, one-tailed p-value = 0.339; n(VEH) = 14, n(OXT) = 14. (B) When tested for anxiety-like behavior in the LDB 5 days after the cOXT infusion ended, % time spent in the LB returned to basal levels in the treatment groups. Data are represented as mean % time spent in the LB ±SEM. n(VEH) = 5, n(1ng cOXT) = 5, n(10ng cOXT) = 5. (C) Anxiety-like behavior of female rats increases in the LDB after 14 days of icv of chronic OXT at a dose of 1ng/h, but not 10ng/h. Data are represented as mean % time spent in the LB. Kruskal-Wallis, H = 7.525, * p = 0.023 vs VEH, n(VEH) = 6, n(1ng cOXT) =7, n(10ng cOXT) = 7. (D) Anxiety-like behavior in the LDB of VEH-treated male rats with and without prior elevated platform stress. Data are represented as mean % time spent in the LB ±SEM. t = 1.812, two-tailed p-value = 0.1075; n = 5.

**Figure S3. Intracellular signaling in the PVN or hippocampus of male and female rats.**

(A) MEK1/2 phosphorylation in the PVN of female rats tended to decrease in the 10ng/h cOXT group without reaching significance. Data are represented as fold changes vs VEH ±SEM. n(VEH) = 4, n(1ng cOXT) = 6, n(10ng cOXT) = 6. (B) Phosphorylation of ERK2 in the PVN of female rats decreased significantly in the 10ng/h cOXT group, whereas ERK1 tended to decrease. No effect on ERK1/2 phosphorylation was observed in the 1ng/h cOT group. Data shown as fold changes vs VEH ±SEM. pERK1: F(2,16) = 3.293, p = 0.067; pERK2: H = 5.795, * p = 0.045; n(VEH) = 4, n(1ng cOXT) =7, n(10ng cOXT) = 3. (C) No significant effect of cOXT treatment on MEF2A DNA-binding in the PVN of virgin female rats has been found. Data are represented as fold changes in DNA binding activity ±SEM F(2;22) = 0.177; p = 0.839; n(VEH/1ng/h cOXT) = 8, n(10ng/h cOXT) = 7. (D) No significant changes in MEF2A and MEF2C binding activity in the hippocampus of male rats have been detected after chronic OXT treatment. Data are represented as fold changes in DNA binding activity ±SEM, indicated by binding of MEF2A *in vitro* to its responsive element and fluorescent antibody-labeling of bound MEF2A. MEF2A: F(20,20) = 0.632, p = 0.543. MEF2C: F(20,20) = 0.347, p = 0.712, all treatment groups = 7. (E) No effects of acute icv OXT infusions have been observed on MEF2A binding activity in the PVN of male rats. Data are represented as mean fold changes (±SEM) of DNA binding activity compared to VEH. Mann-Whitney Rank Sum Test U = 8.000, p = 0.247; n(VEH) = 5, n(OXT) = 6. (F) In contrast to MEF2A (Fig. 2D), MEF2C binding activity increased slightly by 1ng/h, but not 10ng/h cOXT. Data are represented as fold changes in DNA-binding activity ±SEM in the PVN of male rats. F(2;17) = 5.633, p = 0.015, Holm Sidak *p = 0.015 vs VEH; n(VEH) = 6, n(1ng cOXT) = 6, n(10ng cOXT) = 6.

**Figure S4.** **Continuative data in females and chromatin immunoprecipitation analysis.**

(A) Protein ration of mCRFR2α over sCRFR2α expression in PVN tissue lysates of virgin female rats treated with 1 or 10ng/h chronic OXT for 14 days. Unlike in male PVN tissue, no significant differences were detected. (B) Western Blot showing the input control, MEF2A precipitated lysate and antibody-Isotype-control precipitation of the chromatin immunoprecipitation from H32 cells as described in the results section. One single band in the second lane indicates a successful pull-down of MEF2A with cross-linked DNA, which has been analyzed by qPCR using primers covering MEF2A binding sites in Exon 2 and Exon 6. Antibody-Isotype pull down indicates specificity of the MEF2A antibody and is used for background signal subtraction. “Input” serves as loading control for the enrichment by the MEF2A antibody.

**Figure S5. GapmeR and TSB treatment: continuative data.**

(A) Distribution of 5’-FAM-labelled antisense oligonucleotides in the hypothalamic area after microinfusions (1nmol/0.5µl per animal). Direct infusions into the PVN (Bregma -1.7 AP, +/-0.3 ML, -8.2 DV) result in mostly PVN and dorsal hypothalamus staining. Magenta: OXT-Neurophysin positive neurons, green: GapmeRs. (B) Assessment of the anxiolytic effect of the PVN CRFR2α. Anxiety-like behavior of male rats increased in the LDB 10 min after intra-PVN administration of the CRFR2α-specific antagonist antisauvagine-30 (ASV), and decreased following infusion of the CRFR2α-specific agonist stresscopin (SCP). Data are represented as mean ±SEM. F(2;51) = 12.099; p<0.001; Holm-Sidak * p<0.03 vs VEH. n(VEH) = 23, n(ASV) = 21, and n(SCP) = 8. (C) Social preference measured by percentage of time spent investigating (sniffing) a non-social stimulus (empty cage) versus a social stimulus (cage with a conspecific) for 5 min each. All animals display social preference (scr, p = 0.001, GapmeR, p = 0.033, TSB, p<0.01; Bonferroni’s post hoc analysis) with no significant effect of treatment. This indicates that the effect of the sCRFR2α on anxiety-like behavior has no social component.
